# Identification of a Clade-Specific HLA-C*03:02 CTL Epitope GY9 Derived from the HIV-1 p17 Matrix Protein

**DOI:** 10.3390/ijms25179683

**Published:** 2024-09-06

**Authors:** Samuel Kyobe, Savannah Mwesigwa, Gyaviira Nkurunungi, Gaone Retshabile, Moses Egesa, Eric Katagirya, Marion Amujal, Busisiwe C. Mlotshwa, Lesedi Williams, Hakim Sendagire, on behalf of the CAfGEN Consortium, Dithan Kiragga, Graeme Mardon, Mogomotsi Matshaba, Neil A. Hanchard, Jacqueline Kyosiimire-Lugemwa, David Robinson

**Affiliations:** 1 Department of Medical Microbiology, College of Health Sciences, Makerere University, Kampala P.O. Box 7072, Uganda; savannah.mwesigwa@gmail.com (S.M.); hakimsendagire@gmail.com (H.S.); 2 Department of Immunology and Molecular Biology, College of Health Sciences, Makerere University, Kampala P.O. Box 7072, Uganda; katagiryaeric@gmail.com (E.K.);; 3 The Medical Research Council/Uganda Virus Research Institute & London School Hygine Tropical Medicine Uganda Research Unit, Entebbe P.O. Box 49, Uganda; gyaviira.nkurunungi@mrcuganda.org (G.N.); jlugemwa@gmail.com (J.K.-L.); 4 Department of Infection Biology, London School of Hygiene & Tropical Medicine, Keppel Street London, London WC1E 7HT, UK; 5 Department of Biological Sciences, University of Botswana, Gaborone Private Bag UB 0022, Botswana; retshabileg@ub.ac.bw (G.R.); b.mlotshwa@hotmail.co.uk (B.C.M.); williamsl@ub.ac.bw (L.W.); 6 Baylor College of Medicine Children’s Foundation, Kampala P.O. Box 72052, Uganda; dkiragga@baylor-uganda.org; 7 Department of Molecular and Human Genetics, Baylor College of Medicine, Houston, TX 77030, USA; gmardon@bcm.edu; 8 Department of Pathology and Immunology, Baylor College of Medicine, Houston, TX 77030, USA; 9 Pediatric Retrovirology, Department of Pediatrics, Baylor College of Medicine, Houston, TX 77030, USA; matshaba@bcm.edu; 10 Botswana-Baylor Children’s Clinical Centre of Excellence, Gaborone Private Bag BR 129, Botswana; 11 National Human Genome Research Institute, National Institutes of Health, 50 South Drive, Bethesda, MD 20892, USA; neil.hanchard@nih.gov; 12 Department of Chemistry and Forensics, School of Science and Technology, Nottingham Trent University Clifton Lane, Nottingham NG11 8NS, UK; david.robinson@ntu.ac.uk

**Keywords:** HIV control, HLA-C*03:02, immunoinformatics

## Abstract

Efforts towards an effective HIV-1 vaccine have remained mainly unsuccessful. There is increasing evidence for a potential role of HLA-C-restricted CD8^+^ T cell responses in HIV-1 control, including our recent report of HLA-C*03:02 among African children. However, there are no documented optimal HIV-1 CD8^+^ T cell epitopes restricted by HLA-C*03:02; additionally, the structural influence of HLA-C*03:02 on epitope binding is undetermined. Immunoinformatics approaches provide a fast and inexpensive method to discover HLA-restricted epitopes. Here, we employed immunopeptidomics to identify HLA-C*03:02 CD8^+^ T cell epitopes. We identified a clade-specific Gag-derived GY9 (GTEELRSLY) HIV-1 p17 matrix epitope potentially restricted to HLA-C*03:02. Residues E62, T142, and E151 in the HLA-C*03:02 binding groove and positions p3, p6, and p9 on the GY9 epitope are crucial in shaping and stabilizing the epitope binding. Our findings support the growing evidence of the contribution of HLA-C molecules to HIV-1 control and provide a prospect for vaccine strategies.

## 1. Introduction

Host genetic factors play an important role in HIV-1 control [1]. Outside the Δ32 mutation in the *chemokine receptor 5* (CCR5) gene, genome-wide association studies (GWAS) have consistently identified variants in the major histocompatibility complex (MHC, also known as human leucocyte antigens [HLA]) class I alleles to play a significant role in the control of HIV-1 infection [2,3]. The HLA class I alleles predominantly display intracellularly processed viral antigens on cell surfaces to elicit CD8^+^ T lymphocytes (CTL) in the adaptive immune responses [4,5]. This cell-mediated immune response is responsible for the clearance of virally infected cells [4,5]. Therefore, the mechanisms of intracellular antigen processing, including proteasome cleavage, peptide loading, and transportation into the endoplasmic reticulum via the transporter associated with antigen processing and peptide stabilization on the MHC molecule for stable cell surface presentation are a subject of interest to understand the contribution of the HLA class I in viral control [6,7,8,9,10].

We recently documented the putative role of HLA-C*03:02, HLA-B*57:03, and HLA-B*58:01 in long-term non-progression (LTNP) of HIV-1 among children in Uganda and Botswana [11]. In contrast to HLA-B*57:03 and HLA-B*58:01, the specific mechanisms underlying HLA-C*03:02-mediated HIV-1 control have not been fully elucidated. As such, the molecular and immunological basis of how HLA-C*03:02 confers its protective effects against HIV-1 infection remains unknown. Prior to our work, the HLA-C*03:02 allele was demonstrated to have a significant correlation with both reduced viral load and elevated CD4^+^ T cell count within the South African population, although these associations did not attain statistical significance [12]. The frequency of HLA-C*03:02 varies among ethnic groups, with 1–6% in Africans, 1–13% in Asians, and very low (<~0.8%) in most white Western populations, aligning with lower HIV prevalence in those regions [13]. Considering these findings, it is evident that HLA-C*03:02 holds potential significance in HIV control, stimulating further investigation into the mechanisms underlying its immune-mediated effects. HLA-B*57:03 and HLA-B*58:01 molecules display highly restricted HIV-1 epitopes in the structural and non-structural proteins that mediate HIV control [10,14,15,16]. Most of these peptides are derived from the Gag protein; however, high immunogenicity has been demonstrated in the non-structural proteins Nef, Vif, and Vpr [16,17,18,19]. Additional research is therefore needed to unravel the intricate interplay between HLA-C*03:02 and HIV-1 proteins, as well as the immune responses triggered by this particular HLA allele. The HLA-restricted epitopes are characterized by their ability to induce effective qualitative and quantitative cellular immune responses [20,21]. These epitopes are crucial in driving robust and polyfunctional cellular immune responses, contributing to the recognition and targeting of HIV-infected cells [20,21,22,23]. Furthermore, HIV-1 epitopes have been demonstrated to elicit humoral immune responses, generating broadly neutralizing antibodies [24,25]. The selective pressure of protective HLA alleles is known to drive the emergence of escape mutants, though at the expense of viral replication fitness, which other compensatory mutations may counter [14,26]. However, the accumulation of escape mutations in HLA-B*57:03/B*58:01-restricted epitopes abrogates the protective effect through various mechanisms, including qualitative binding to killer immunoglobulin-like receptors [14,27]. Nonetheless, developing epitope-based vaccines that efficiently elicit both humoral and cellular immune responses has re-emerged as a strategy to control the global HIV-1 epidemic [5,28,29].

The success of multi-epitope HIV-1 vaccines remains generally challenging due to the rapid genetic evolution of the virus, diverse HLA genetic polymorphism, and viral-clade geographical diversity [11,30,31]. Previous research has predominantly focused on characterizing HLA-restricted epitopes specific to protective HLA-A and HLA-B alleles in the context of HIV. At the same time, comparatively limited consideration has been given to exploring the protective HLA-C alleles [10]. Therefore, identifying and prioritizing protective HLA-C-restricted epitopes from the locally prevalent HIV-1 clade remains viable for designing an optimal vaccine candidate. The scientific literature presents many methodological approaches for identifying optimal HIV-1 CTL epitopes, each yielding diverse outcomes [15,32]. This diversity underscores the complexity of epitope prediction and necessitates careful consideration of the most appropriate methodologies for accurate and comprehensive epitope discovery. However, it is crucial to emphasize that these approaches consistently exhibit a strong agreement between predictive and experimental methods [33]. In this study, we employed an immunoinformatics approach and identified four potentially HLA-C*03:02-restricted CD8^+^ T cell epitopes. Furthermore, using an ELISpot assay, we experimentally validated that a clade-specific GY9 epitope derived from the p17 HIV-1 matrix protein is potentially restricted to HLA-C*03:02 alleles in an African (Ugandan) population. Our observations further support the growing evidence of the contribution of HLA-C molecules to HIV-1 control and provide an opportunity for innovative vaccine strategies.

## 2. Results

### 2.1. HIV-1 Clades C and A Have Private and Shared HLA-C*03:02-Epitopes and Preferentially Accommodate Hydrophobic Residues in the Distal Pocket

**Figure 1:**
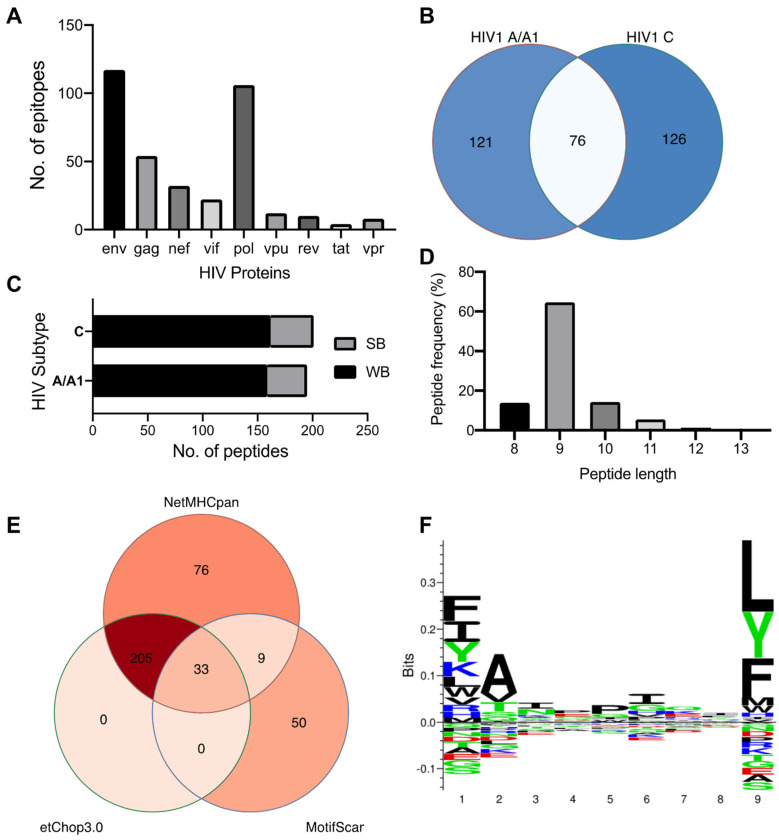
HIV 1 subtypes A/A1 and C predicted epitopes. (**A**) Distribution of epitopes predicted as strong or weak binders by HIV protein (n = 323). (**B**) Venn diagram showing the number of shared predicted epitopes between HIV-1 subtype A/A1 and C. (**C**) Proportion of HIV 1 predicted to be strong or weak binders by HIV subtype (A/A1 = 197, C = 202). (**D**) Frequency of the length of predicted peptides (n = 323). (**E**) Venn diagram showing the number of peptides predicted by NetMHCpan and Motif Scan methods. Also, the number of epitopes predicted to undergo proteasomal cleavage by TAP is shown. (**F**) Amino acid sequence logo representation of the most abundant residue at each position in the epitope (n = 323). Prominent amino acid symbols indicate the most preferred amino acid in that epitope position. The sequence logo was calculated using clustering and pseudo counts with a weight on prior at 200, and data was handled with probability-weighted Kullback–Leibler probability distribution. Abbreviations: SB, strong binders; WB, weak binders.

We used the NetMHCpan 4.1 and MotifScan servers to predict the epitope repertoire of HLA-C*03:02, and a total of 42,679 and 92 peptides were predicted using HIV-1 clade A/A1 and C proteins, respectively. Expectedly, the *env* and *pol* genes contribute the largest number of epitopes (Figure 1A). Among the NetMHCpan-predicted epitopes, 75 and 321 were predicted to meet the strong and weak binders’ threshold, respectively (Table S1). The thresholds are expressed in terms of %Rank, the percentile of the predicted binding affinity compared to the distribution of binding affinities calculated on a set of random natural peptides. A similar number (and proportion) of strong and weak binders were predicted from the HIV-1 A and C proteome, and 76 (23.5%) peptides were found to be shared among the clades (Figure 1B,C). We then used NetChop 3.0 to determine a final set of 238 epitopes predicted to undergo proteasomal cleavage (Figure 1D). These epitope sequences range from 8 to 13 mers with a predominance of 9 mers (65%, Figure 1E). Further analysis of their amino acid sequence pattern at the HLA-C*03:02 motif using sequence logos (Figure 1F) found that certain amino acids are predominant or conserved at positions 1 (p1), 2 (p2), and 9 (p9, C-terminus). The P9 position of the HLA-C*03:02 motif is occupied by leucine, a large hydrophobic amino acid, but the position also accepts large hydrophobic and neutral residues phenylalanine and tyrosine, respectively (Figure 1F). At position p2, the small hydrophobic residue alanine is preferred, but the small hydrophobic and neutral residues valine and threonine, respectively, are also accommodated. Similar to p9, position p1 equally favors large hydrophobic residues phenylalanine and isoleucine, and a large hydrophilic lysine, but also accepts a large neutral residue, tyrosine (Figure 1F).

### 2.2. C*03:02-Restricted Stable Epitopes Are Mainly Derived from Structural Proteins of HIV

**Figure 2:**
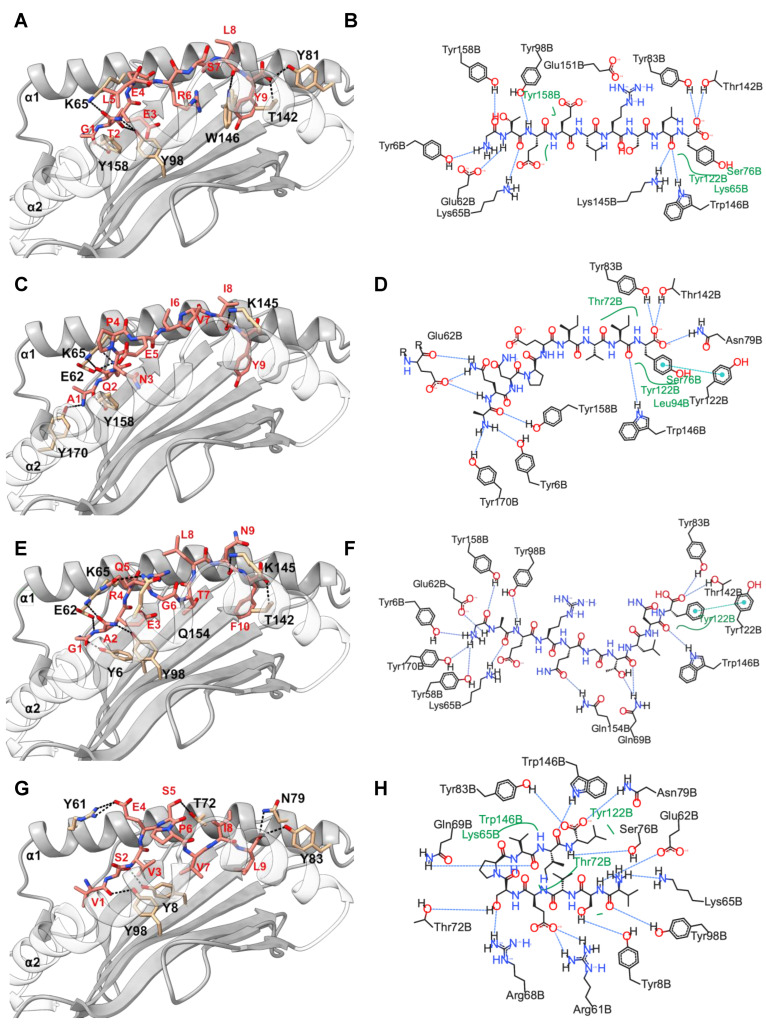
Molecular docking analysis and representation of HLA-C*03:02 interactions with docked epitopes GY9 (**A**,**B**), AY9 (**C**,**D**), GF10 (**E**,**F**), and VL9 (**G**,**H**). In the left panel is a 3D depiction of the HLA-C*03:02 molecule (ribbon representation) alongside the epitope (stick representation). Dashed lines highlight the presence of hydrogen bonds. Residues within HLA-C*03:02 contributing to hydrogen bond interactions are labeled in black, and residues in the epitope are labeled in red. The α2 chain has been rendered transparent, enabling clear visualization of the epitope. In the left panel is a 2D depiction (generated using PoseView, https://proteins.plus/ accessed on 15 November 2022) of the docked epitopes, with hydrogen bonds depicted as black dashed lines and van der Waals forces shown in green. These illustrations provide an insightful view of the molecular interactions.

**Figure 3:**
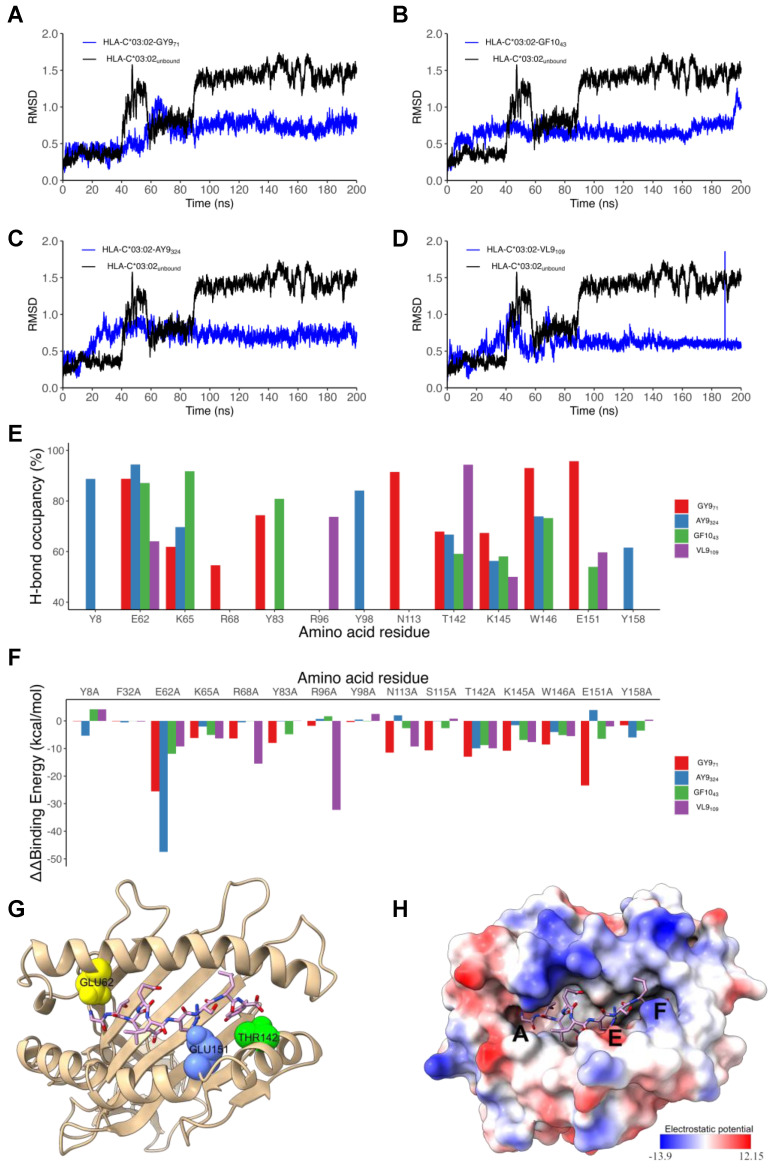
Molecular dynamics simulations analysis of HLA-C*03:02/peptide complexes. (**A**–**D**) are RMSD plots of the Cα backbone of HLA-C*03:02 in complex with VL9 **(A)**, GY9 (**B**), GF10 (**C**), and AY9 (**D**). (**E**) The percent of hydrogen-bond occupancy for interactions between HLA-C*03:02 residues (donors or acceptors) and stable epitopes (VL9, GF10, GY9, and AY9) across the final 100 ns. (**F**) Binding free energy change of key residues involved in HLA-C*03:02 binding with epitopes. (**G**) A 3D structure of HLA-C*03:02 displaying the key residues Glu62, Glu151, and Thr142 in pocket A (yellow), E (blue), and F (green), respectively. (**H**) The electrostatic surface potential of HLA-C*03:02 Electrostatic potential was calculated and visualized using ChimeraX default settings. The color scale ranges from −13 (red) to +12 (blue) kT/e.

Next, we performed in silico docking to determine and characterize the top-ranked HLA-C*03:02 epitopes that preferentially elicit CD8^+^ T cell responses accounting for the putative protective effect [11]. First, we designed a 3D structural model of the HLA-C*03:02 molecule. The best template for model building was protein data bank (PDB) ID 5w6a.2 (HLA-C*06:02), with high sequence identity (94.7) and coverage, resulting in a model with high confidence scores, favorable stereochemistry, and stability, suitable for ligand binding studies (molecular docking) (Figures S1 and S2, Table S2). According to our docking protocol, we found eight top-ranked conformations (peptides); with the best energetically favored docking scores and extensive strong peptide-HLA (pHLA) hydrogen bonds (Figure 2A–D and Figure S3, Table 1). Four epitopes were found in structural HIV-1 proteins, including ^71^GTEELRSLY^79^ (GY9) located on the *gag* gene derived from the p17 matrix protein, ^43^GAERQGTLNF^52^ (GF10), and ^324^AQNPEIVIY^332^ (AY9) encoded on the *pol* gene and ^58^KAYETEMHN^66^ (KN9) located in the *env* gene derived from the gp120 protein. Other epitopes were derived from non-structural HIV-1 proteins, such as ^84^GAFDLSFFL^92^ (GL9) and ^114^WVYNTQGYF^122^ (WF9), from the Nef protein, while ^128^VVSPRCEY^135^ (VY8) and ^109^VSVESPVIL^117^ (VL9) are derived from Vif and Rev proteins, respectively. To establish the structural basis of the stability of these predicted pHLA complexes, we performed an extensive conventional molecular dynamic (MD) simulation. We performed all-atom MD simulations of HLA-C*03:02 in the unbound form and on each of the eight pHLA complexes. The root means square deviation (RMSD) of protein atoms from their initial structural position over time provides an assessment of the stability of the protein–ligand complexes. We calculated and compared the average RMSD of the Cα atoms of the pHLA complexes and the unbound HLA-C*03:02 (1.05 Å). Four pHLA complexes exhibited convergence, particularly evident within the final 100 ns of MD, as depicted in Figure 3A–D. The achieved convergence is reflected in notably reduced average RMSD values: 0.66 Å (GY9), 0.67 Å (GF10), 0.70 Å (AY9), and 0.59 Å (VL9) compared to the free HLA-C*03:02 molecule (Figure 3A–D). The remaining pHLA complexes with KN9, GL9, WF9, and VY8 epitopes exhibited a lack of stability (Figure S3G,H). In our subsequent molecular dynamics (MD) trajectory analyses, we focused on analyzing the last 100 nanoseconds. This data indicate that the molecules GY9, GF10, AY9, and VL9 exhibit enhanced conformational stability of the HLA-C*03:02 molecule upon binding.

We then examined the formation of intermolecular interactions of the stable pHLA complexes. Hydrogen bonds play a significant role in forming and stabilizing pHLA complexes in the binding groove of HLA-C*03:02. We examined the hydrogen bond occupancy between the four epitopes and HLA-C*03:02 using the hydrogen bond module in VMD software [34]. We analyzed strong hydrogen bonds with an acceptor–donor atom distance ≤3.5 Å and a hydrogen-to-donor-acceptor angle greater than 120°. We observed that residues Glu62, Thr142, and Lys170 in HLA-C*03:02 were involved in hydrogen bond formation with all four epitopes with more than 50% occupancy (Figure 2 and Figure 3E, Table S3). More than 50% hydrogen bond occupancy existed between Lys90, Trp171, and Glu151 in HLA-C*03:02 with at least three of the four epitopes (Figure 3E, Table S3). These results suggest that the HLA-C*03:02 binding groove favorably and stably binds three epitopes derived from structural HIV-1 proteins.

### 2.3. Positions 62, 142, and 151 in HLA-C*03:02 and P6 in the GY9 Epitope Provide the Structural Basis for the Preferential Binding of GY9

The binding free energy (ΔG) of pHLA complexes determines the stability of complex formation. Therefore, we applied the molecular mechanic/Poisson–Boltzmann surface area (MM/PBSA) method to estimate the binding free energies of GY9, AY9, GF1,0, and VL9 complexation with HLA-C*03:02. Generally, a more negative magnitude of the binding free energy corresponds to strong (high) binding affinities of pHLA complexes. Among the epitopes, GY9 showed a much stronger binding free energy of −88.41 kcal/mol, indicating a strong and favorable binding affinity to HLA-C*03:02 (Table 2). Notably, the van der Waals and electrostatic energies of −53.01 kcal/mol and −547.44 kcal/mol, respectively, between GY9 and HLA-C*03:02 contribute significantly to the binding (Table 2). We found that the electrostatic contribution of the GY9 epitope is much higher compared to other epitopes. Given the significant contribution of hydrogen bonding formation to the electrostatic energy, this means that hydrogen bonds are likely to play a critical role in GY9 binding to HLA-C*03:02. Also, the van der Waals energy is an indicator of the compactness of a ligand in the receptor binding groove; we found that GY9 also had the strongest value (−53.01 kcal/mol), suggesting a more favorable packing arrangement of GY9 in the HLA-C*03:02 binding groove (Table 2). This compactness is essential in pHLA complexes and affects efficient T cell receptor (TCR) engagement [35,36]. Overall, these findings provide valuable insights into the importance of van der Waals interactions, electrostatic interactions, and hydrogen bonding in the binding dynamics of HIV-1 epitopes to HLA-C*03:02, as well as the preference for GY9.

To gain insight into the individual contributions of the amino acids within the HLA-C*03:02 binding groove to the binding free energy, we performed a computational alanine scanning (CAS, or mutagenesis) based on the MM/PBSA method [37]. A negative value of ΔΔG indicates a favorable contribution for the wild-type residue in that position and vice versa. We mutated 35 amino acid residues within 5 Å of the epitopes to alanine and computed the binding free energy difference between wild-type and mutant pHLA complexes. Notably, mutants E62A, T142A, and E151A in HLA-C*03:02 resulted in a significant loss of binding free energy with GY9 (Figure 3F, Table S4). For position 62, the mutation to alanine (E62A) resulted in a loss of binding free energy ranging from −9.25 kcal/mol to −47.47 kcal/mol across different peptide ligands (GY9, GF10, AY9, VL9). Similarly, for position 142, alanine mutation (T142A) led to a decrease in binding free energy ranging from −9.91 kcal/mol to −9.94 kcal/mol. Likewise, for position 151, alanine mutation (E151A) resulted in reduced binding free energy ranging from −1.98 kcal/mol to −23.40 kcal/mol, except for AY9, where a positive change in binding free energy of 3.93 kcal/mol was observed. Consequently, these three positions, E62, T142, and E151, found in the A, E, and F pockets of the HLA-C*03:02 peptide-binding groove (Figure 3G), are predicted to confer epitope specificity. However, G1 on GY9 could not be alanine scanned due to the limitation of alanine scanning, it should be noted that glycine and alanine share similar properties.

Previous studies have shown that point mutations within epitopes significantly diminish or abrogate immune responses [36]. We performed CAS on the GY9 epitope to establish the most influential positions to the binding affinity. Surprisingly, we noted a consistent trend in a decrease in the binding free energy (ΔΔG) across all the amino acid positions in GY9 except p1 with a small glycine residue (similar to alanine). However, p6 demonstrated a significant negative loss in binding free energy (ΔΔG −27.00 kcal/mol; Table 3), further reinforcing the importance of Arg6 at this position for binding affinity. We observed that Arg at position p6 in GY9 leads to the forming of three hydrogen bonds (donor) with residues E176, W171, and N138 (Table S3). Remarkably, these hydrogen bonds exhibit a high occupancy of over 90% throughout the MD simulation, indicating their persistent and stable nature. This suggests that stabilizing p6 is vital to prevent protrusion of the epitope out of the peptide binding groove that would considerably alter the structure of the pHLA-TCR binding platform. We computed the conservancy score by aligning viral sequences from all publicly available HIV-1 subtypes A, C, D, and K and their recombinants. We also found that p3, p6, and p9 had the lowest conservancy score (Table 3). These results suggest that p6 contributes favorably to GY9 binding and may serve as the primary anchor residue, while positions p3 and p9 are secondary anchor residues refining epitope binding.

### 2.4. The GY9 Epitope Elicits a Clade-Specific HLA-C*03:02 IFN-γ Response

**Figure 4:**
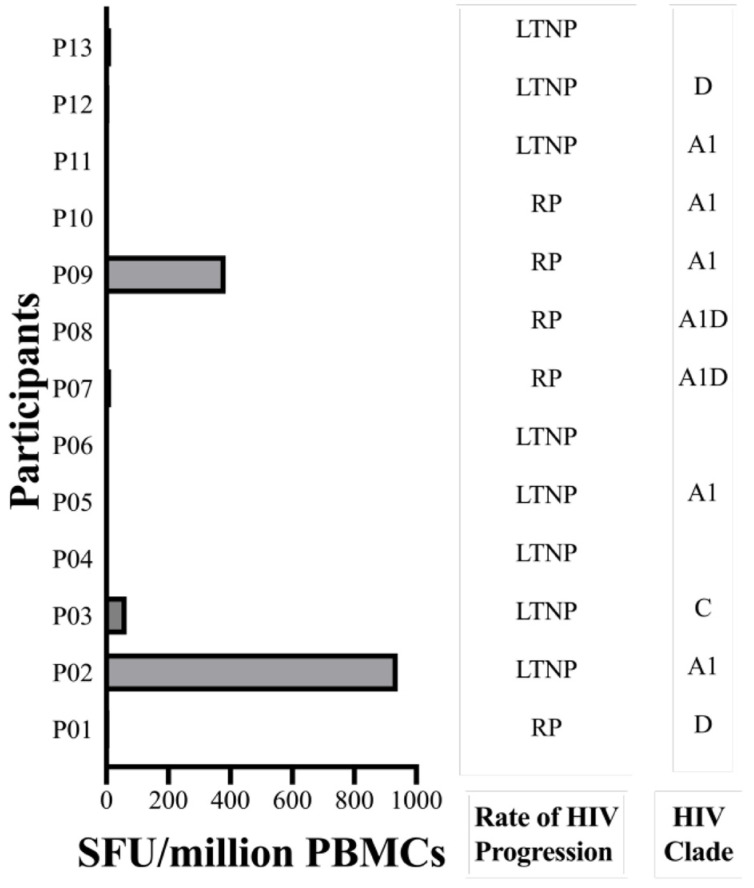
GY9-specific IFN-γ CD8^+^ T cell responses among HLA-C*03:02^+ve^ participants. The magnitude of IFN-γ CD8^+^ T cell responses against GY9 epitope was measured among HLA-C*03:02^+ve^ and HLA-C*03:02^−ve^ (Table S5) participants by ELISpot assay. LTNP, long-term non-progression; RP, rapid progression Note: P06, P08, and P10 did not have sufficient cells.

To discern the immunogenic potential of GY9 ex vivo, we assessed GY9-specific CD8^+^ T cells, employing a dual color enzyme-linked immunospot (ELISpot) assay to measure the production of IFN-γ and IL-2. IFN-γ production indicates an active immune response, reflecting ongoing T cell effector functions. On the other hand, IL-2 secreted by activated T cells or NK cells plays a crucial role in driving the proliferation and differentiation of naive T cells, B cells, and NK cells, facilitating their transition into effector (such as Th1) and memory cells, and promoting the release of secondary cytokines. We used peripheral blood mononuclear cells (PBMC) from a study population that included 25 subjects on antiretroviral therapy (ART) recruited from Uganda, of whom 13 expressed the HLA-C*03:02 allele (HLA typing is described elsewhere, Table S5) [11]. GY9-specific IFN-γ production ranging from 65 to 940 SFU/million PBMC, was found in 3/10 (30%) HLA-C*03:02^+ve^ subjects (with PMBC available). Still, no response was found among any HLA-C*03:02^−ve^ subjects (Figure 4, Table S5). However, the difference in ELISPOT responses between HLA-C03:02^+ve^ and C*03:02^−ve^ individuals did not reach statistical significance (*p* = 0.078, Fisher’s Exact test). All GY9 responders were coincidentally infected with HIV-1 clade A1 (2/3) or C (Figure 4, Table S5). It should be noted that GY9 originated from both the A1 and C clade consensus sequences (https://www.hiv.lanl.gov/content/index accessed on 30 January 2022). Except for three individuals for whom HIV-1 could not be typed, all non-responders to GY9 were found to be infected with HIV-1 clades A1, C, D, or A1D recombinant strains (Table S5). Unsurprisingly, in our cohort on chronic ART (1–121 months), we detected no IL-2 production in HLA-C*03:02^+ve^ or HLA-C*03:02^−ve^ individuals (Table S5) [38]. We have already demonstrated above that some positions with the GY9 epitope are under selective pressure (CAS and conservancy scores). We think the lack of response in HLA-C*03:02^+ve^ subjects infected with A1 may suggest the presence of escape mutants, especially in positions p6 > p3 > p9. These findings indicate that GY9 elicits a clade-specific immune response and exhibits non-promiscuity for HLA types.

## 3. Discussion

Several approaches are being investigated to develop novel HIV-1 vaccines. Among these approaches is the search for multi-epitope vaccine candidates that elicit effective quantitative and qualitative humoral and cellular immune responses [28,39]. Theoretically, T-cell-based vaccines, utilizing peptides identified through in silico predictions, hold promise as effective vaccination strategies, particularly when focused on pinpointing the most immunogenic antigens [28]. In this study, we have utilized a synergy of computational techniques and empirical functional validation to uncover a previously unrecognized HIV-1 epitope, GY9. This epitope displays a distinctive potential for presentation by the HLA-C*03:02 allele, associated with effective HIV-1 control among African pediatric populations. Consistent with previous reports, the role of the HIV-1 Gag protein is prominent in providing the most immunogenic peptides presented by class I HLA molecules. Additionally, we report three potential epitopes in the Env and Pol proteins that map to HIV-1 subtypes A and C, suggesting that some control of HIV-1 may be attributable to HLA-C*03:02.

While previous research on HIV-1 vaccine candidates has predominantly focused on the protective HLA-B alleles, it is noteworthy that HIV-nef attachment selectively downregulates the cell surface expression of both HLA-A and B molecules [40,41]. This downregulation phenomenon facilitates immune evasion through CTL escape by virally infected cells. Consequently, HLA-C-restricted CTL responses remain intact to facilitate the recognition and destruction of HIV-infected cells. Most crucially, the HLA-C*03:02 cytoplasmic tail lacks both tyrosine and aspartate, which are the targets of Nef-dependent downregulation of HLA cellular surface expression. Instead, HLA-C*03:02 has Leu321 and Val328 in the cytoplasmic tail [40]. Therefore, compensatory mechanisms enhance HLA-C cell surface expression, favorably explaining the role of HLA-C-restricted CTL responses in HIV-1 control [42]. Furthermore, HLA-C alleles lacking a binding site for microRNA-148a in the 3′ untranslated region of their messenger RNA exhibit a compensated high surface expression, potentially influencing immune recognition and responsiveness [43]. Interestingly, the HLA-C*03:02 allele demonstrates strong linkage disequilibrium with a C variant located 35kb upstream of the HLA-C gene. The presence of the -35C allele is strongly associated with increased cell surface expression of HLA-C molecules, potentially providing a mechanistic explanation for the observed impact of HLA-C*03:02 on HIV-1 control [42,44]. In this study, we find that GY9-induced IFN-γ responses were not shared with other HLA-C, -A, or -B alleles (Table S5); this would suggest that clade-specific GY9 HLA-C*03:02-restricted responses are potentially allele specific. This potentially restricted binding specificity of the GY9 epitope is predicted to play a crucial role in determining immune responses following HIV-1 infection and may have implications for vaccine design and understanding the individual variation in immune recognition.

The HIV-1 Gag protein is preferred for T cell vaccine candidates because it is highly immunogenic and conserved across HIV-1 clades [17,45]. Several T cell candidate vaccines have so far shown variable immunogenicity [45]; however, thus far, this GY9 epitope has not been reported to show immunogenicity or potential restriction to HLA-C*03:02 and therefore has not been considered a potential vaccine candidate [45,46]. This could be attributed to the lack of prioritization of HLA-C preferential antigens. The role of HLA-C class I molecules in delaying HIV-1 progression has been historically considered less significant, primarily attributed to their lower cellular surface expression levels and high LD with HLA-A and B alleles. Consequently, their contribution to HIV-1 control has not been prominently emphasized. Notably, to date, less than 10% (22/280) of optimal HIV-1 CTL epitopes (“*A list*”) defined in the LANL HIV-1 epitope database are HLA-C-restricted epitopes. Accumulating evidence underscores the potential role of HLA-C molecules in HIV-1 control, especially in relation to conserved Gag protein [32,47,48]. In our study, we observed a variable magnitude of IFN-γ responses and no detectable IL-2 response upon stimulation of T cells from people living with HIV with the GY9 epitope, which is consistent with findings reported in previous studies [48,49]. This variable response could be attributed to several factors, such as immune exhaustion due to chronic infection and ART (for IL-2 ablation and low IFN-γ production) among this cohort and viral escape within the GY9 epitope (for no IFN-γ responses) [48,50,51,52]. Indeed, we measured the magnitude of response using study participants on ART (average duration 32 months (1–121 months)) [52].

A striking absence of both IFN-γ and IL-2 responses was observed in a larger number of participants with the HLA-C*03:02 allele; this aligns with the likelihood of amino acid mutations within the GY9 epitope sequence. Indeed, when we calculated conservancy scores, p6 and p3/p9 had a very low score (2), which means that these positions are associated with a high rate of mutations across the various HIV-1 clades A, C, D, and K. Similarly, our CAS studies detected significant differences in the pHLA relative binding free energy where residues in p6 and p3/p9 are mutated to alanine, suggesting a very high contribution to epitope binding. A recent report by Li et al. suggests that mutations within the epitope significantly impact pHLA binding due to conformational changes and eventually affect TCR recognition and antigen presentation [36]. Collectively, our data indicates that p6 and p3/p9 within the GY9 epitope potentially serve as primary and secondary anchor residues, respectively. These are important for binding within the HLA-C*03:02 antigen-binding cleft, thereby facilitating optimal T cell receptor (TCR) engagement. Indeed, Joglekar et al. demonstrated that peptide-MHC binding is essential for TCR binding and that peptide mutations play an important role in viral escape [53]. Therefore, we argue that these findings show a potential viral escape and immune evasion pathway within the GY9 epitope [36]. The absence of detectable IL-2 responses to the GY9 epitope underscores the impaired capacity to reactivate HIV-specific memory T cells elicited during chronic infection, indicating a compromised immune response [38]. This observation aligns with the known phenomenon that HIV-1 infection leads to an expansion of CD8^+^CD28^−^ T cells, characterized by their compromised ability to produce IL-2 [54]. Our data shows that residues E62, T142, and E151 in the HLA-C*03:02 binding groove, along with positions p3, p6, and p9 on the GY9 epitope, are hot spots for binding. These residues play a role in shaping and stabilizing the protein-protein interface, significantly contributing to its stability.

Immunogenicity in HIV-1 is not restricted to the Gag protein since numerous studies have established the role of epitopes derived from other HIV-1 proteins [32]. Indeed, HIV-1 vaccine candidate studies have demonstrated an advantage of multi-epitope prototypes [45]. In this study, our immunoinformatic approach identified three potentially immunogenic epitopes, GF10/AY9 and VL9, derived from the Pol and Rev proteins, respectively; however, we did not find any detectable HIV-1-specific CD8^+^ T cell responses against these epitopes in our African (Ugandan) population. While the lack of responses could be explained by similar factors noted above, the epitopes GF10 and AY9 are derived from the HIV1-C subtype; all our HLA-C*03:02^+ve^ participants used for the dual IFN-γ/IL-2 ELISpot assay were infected with HIV1-A1, C, D and the A1D recombinant. When we performed a conservancy score, we found that many positions along the epitopes had a very low conservancy score (VL9 > GF10 > AY9) that could explain these positions as escape mutations that abrogate responses to epitopes derived from other HIV-1 clades and the potential unsuitability of these epitopes [36]. Overall, our docking results are similar to an experimental biological study where only 6-8 HIV-1 derived peptides were identified as restricted to HLA class I alleles [55]. In that study, Ziegler et al. infected CD4^+^ T cells with HIV-1 and measured HLA class I (HLA-A*02:01/*02:01, B*27:05/*40:01, C*02:02/*03:04) repertoire, suggesting that these molecules present a small set of epitopes derived from the HIV-1 proteome at variable relative quantities [55].

### Limitations of the Study

Our study has some notable limitations. First, performing multiple experiments (replicas) helps ensure the reliability and reproducibility of results. However, in all-atom MD simulations, running replicas is computationally expensive and multiple studies have instead performed longer MD simulation studies. While longer simulations may seem more impressive, recent analyses suggest that using multiple shorter/longer replicas is better for reproducibility and reliability. In particular, Knapp et al. [56] reported that multiple replicas, as opposed to relying on single MD simulations, enhance result reproducibility and reliability in pMHC MD studies. But, there is still a lot of variation in how many replicas should be conducted [56,57]. Therefore, given this limitation, the results of our single all-atom MD study despite achieving a reasonable convergence should be interpreted cautiously. We strongly recommend that future MD studies should include replicas including exploring methods such as coarse-grained methods that can significantly limit simulation times [58]. Secondly, while responses to GY9 were noted among HLA-C*03:02^+ve^ individuals, the study’s statistical power is compromised by the limited sample size. Consequently, our analysis primarily offers a descriptive examination, emphasizing the imperative of a larger sample for the validation and substantiation of our findings. Finally, full or partial viral sequencing and CD4^+^ T cell counts were not performed on all participants in our study. Full viral genome sequencing could have yielded valuable insights into the degree of conservancy within the GY9 sequence among individuals positive or negative for the HLA-C*03:02 allele, enabling empirical assessment of epitope dominance in HIV-infected individuals. Our use of consensus and primary strain sequences for epitope prediction may potentially overlook naturally occurring epitopes in the studied population. A comprehensive analysis of primary strain sequences is crucial to identify conserved epitopes capable of eliciting robust and broad immune responses. Nonetheless, a recent study by Bugembe et al. demonstrated that the same computational tools used here identify 95% of experimentally mapped HIV-1 clade A and D epitopes [33]. Furthermore, measurements of CD4^+^ T cell levels would have provided a baseline assessment of immunocompetence, a factor known to influence immune responses to HIV-1 epitopes [59]. In the future, investigations employing well-characterized study populations, incorporating advanced immunopeptidomics techniques, intracellular cytokine flow cytometry, and tetramer staining assays will be essential to build upon our current findings and overcome the methodological limitations observed in our study [32,60]. These approaches hold the potential to deepen our understanding of immunological responses and contribute valuable insights to the field of immunology.

## 4. Materials and Methods

### 4.1. Patient Recruitment

We used stored PBMC samples from 25 previously recruited participants in the parent study: the Collaborative African Genomics Network (CAfGEN). The details of participant recruitment have been described in detail elsewhere [11,61]. The clinical characteristics of patients before and after treatment are presented in Table S5. We selected all 13 participants expressing the HLA-C*03:02 allele and 12 controls that are HLA-C*03:02^−ve^.

### 4.2. HLA-C*03:02 Homology Modeling and Validation

The 3D structure model of HLA-C*03:02 was predicted using SWISS-MODEL (https://swissmodel.expasy.org/ accessed on 9 October 2020), starting with the 366 amino acids full-length protein sequence downloaded from the IMGT/HLA database (https://www.ebi.ac.uk/ipd/imgt/hla/ accessed on 9 October 2020). An optimal template to model the HLA-C*03:02 protein was selected based on PDB ID: 5w6a.2 HLA-C*06:02 with a sequence identity >90%, query coverage ≥70%, and X-ray resolution at ≤2 Å. The constructed model underwent comprehensive validation assessments in two distinct stereochemical and spatial analysis domains. The stereochemical analysis of parameters, including bond length, torsion angle, and rotational angle, within the model was evaluated using online tools servers SAVES (https://saves.mbi.ucla.edu/ accessed on 15 November 2020) and Pro-Q scores (https://proq.bioinfo.se/cgi-bin/ProQ/ProQ.cgi accessed on 15 November 2020). The Ramachandran plot confirmed stereochemical quality (https://www.ebi.ac.uk/thornton-srv/software/PROCHECK/ accessed on 3 December 2020). The spatial features of the model based on the 3D conformation were analyzed using the Verify 3D (https://saves.mbi.ucla.edu/ accessed on 15 November 2020) and ProSA scores (https://prosa.services.came.sbg.ac.at/prosa.php accessed on 15 November 2020). The model’s overall quality was determined from the ProTSAV score (http://www.scfbio-iitd.res.in/software/proteomics/protsav.jsp accessed on 7 March 2021).

### 4.3. HIV-1 Ligand Prediction and Preparation for Docking

HIV-1 ligands (8-14 mer) predicted to bind HLA-C*03:02 were determined using NetMHCpan-4.1b server (https://services.healthtech.dtu.dk/services/NetMHCpan-4.1/ accessed on 13 May 2021) and supplementary epitopes with Motif Scan (https://myhits.sib.swiss/cgi-bin/motif_scan accessed on 13 May 2021). We used full-length HIV-1 subtype A/A1 and C consensus sequences retrieved from the HIV-1 Sequence Database (https://www.hiv.lanl.gov/content/index accessed on 13 May 2021) representative of the predominant circulating clades in Uganda and Botswana, respectively [62]. From the available ligands, we selected a subset of those predicted as strong or weak binders by NetMHCpan-4 or Motif Scan. Additionally, these ligands were predicted to undergo proteasomal cleavage according to NetChop v3.1 (https://services.healthtech.dtu.dk/services/NetChop-3.1/ accessed on 12 May 2021). For the docking experiment, the 3D structure of the selected linear peptides was predicted using the PEP-FOLD3 server (https://mobyle.rpbs.univ-paris-diderot.fr/cgi-bin/portal.py#forms::PEP-FOLD3, accessed on 19 August 2021) followed by an energy minimization step using the minimize structure module in Chimera [63]. Briefly, essential hydrogens were added, and Gastiger charges were assigned to ligand residues using the Amber ff14SB force field. The 3D structures achieved convergence after 100 steps of steepest descent followed by 1000 steps of conjugate gradient [63].

### 4.4. Molecular Docking Protocol and Analysis

The model HLA-C*03:02 structure was used for docking HIV-1 ligands with DINC, a parallelized meta-docking method for the incremental docking of large ligands. Some modifications were adopted to the default DINC protocol [64]. The grid box of 50 × 40 × 72 xyz points with a grid spacing of 0.375 Å was generated and centered at 11.95 × 57.95 × −6.34 around the six binding pockets using AutoDock Tools [65]. To maximize the docking accuracy, the vina exhaustiveness was set to 8, and the number of binding modes generated at each round of incremental docking was set at 40. An additional round of docking was performed using the whole ligand with full flexibility to obtain a larger docking sampling. The predicted ligand poses were rescored using Convex-PL, shown to achieve >80% accuracy in identifying the best binders [66]. Molecular visualization with UCSF ChimeraX was used to identify and analyze the intermolecular (pHLA) interactions [63,67]. The selection of top-ranked ligand poses was guided by several rigorous criteria, including a Convex-PL score ≥ 7, a DINC binding score ≤ −7.0 kcal/mol, a minimum of 6 strong hydrogen bonds formed with the pHLA complex, and an RMSD ≤ 1.7 Å compared to the native ligand (PDB ID: 5w6a.2) of the C*0602 allele.

### 4.5. Molecular Dynamics Simulation Protocol and Analysis

MD simulations were performed on the top-ranked ligands using GROMACSv2020.3 software under the CHARMM36 all-atom force field [68,69]. The receptor–ligand coordinates generated during molecular docking were utilized to reconstruct protein–ligand complexes using Chimera. All hydrogen molecules were removed from the final structure. We used the Avogadro program to add hydrogens to ligands and the CHARMM36m program to generate ligand parameters and topologies [70,71,72,73]. The resultant HLA-C*03:02-unbound and HLA-C*03:02-ligand complex were solvated in the center of a cubic unit cell of the volume of 10,000 nm^3^ with ~31,000 molecules of TIP3-point water. We allowed a minimum distance of 1 nm between the box boundary and the complex. The system was neutralized with the addition of 10 Na^+^ ions. The system was subjected to energy minimization using the steepest descent method with a maximum force constraint of 10 kJ/mol. Position restraints were applied on both the ligand and HLA-C*03:02 receptor. The system temperature and pressure were equilibrated at 300 K using the modified Berendsen thermostats coupling method and at 4.5 × 10^−5^ bar^−1^ using the Berendsen coupling barostats method, respectively, for 1000 ps. All relaxed systems were subjected to MD simulations for 200 ns using periodic boundary conditions without ligand–protein restraints. The stability of the complexes was examined by analyzing changes in the root mean square deviation (RMSD) and hydrogen bonds network using GROMACS functions hbond and rms, respectively [68].

The binding free energy (denoted as ΔG_bind_ = ΔG_complex_ − ΔG_receptor_ − ΔG_peptide_) was calculated using the molecular mechanics (MM) with Poisson–Boltzmann (PB) and surface area solvation method implemented in the gmx_MMPBSA program [37]. The critical residues at the interface of pHLA binding (within 5 Å of the ligand) were determined by performing computational alanine scanning (CAS) experiments on the ligand and HLA-C*03:02. The resultant binding free energy due to the mutant residue was calculated by comparing the wild-type (ΔG_wild-type_) and mutant (ΔG_mutant_) complexes, as denoted by the equation: ΔΔG_bind_ = ΔG_wild-type_ − ΔG_mutant_.

### 4.6. HIV-1 Epitope Conservancy Analysis

To assess the positional conservancy of the candidate epitopes at the individual residue level, we used the AL2CO sequence conservation analysis server (http://prodata.swmed.edu/al2co/, accessed on 11 May 2023). Specifically, we utilized an alignment file generated from African representative HIV-1 clades A, C, D, and K and their recombinant sequences deposited in the LANL HIV-1 Sequence Database (https://www.hiv.lanl.gov/content/index, accessed on 11 May 2023) for calculating the conservancy scores.

### 4.7. HIV-1 Genotyping

The participants’ genomic DNA was extracted from whole blood with the PaxGene DNA blood kit (Qiagen, Hilden, Germany) as previously described. A three-round nested PCR assay was performed targeting the HIV-1 proviral DNA 712 bp (HXB2 location 2610-3322) Gag-Pol region (the third round nested PCR is to add Illumina-specific adaptor sequences, HXB2 location 2796-3271) [74,75]. The final PCR product was purified using the Agencourt AMPure XP magnetic beads (Beckman Coulter, Brea, CA, USA). The purified PCR was used for library preparation using the Nextera XT DNA Library Preparation Kit (Illumina, San Diego, CA, USA) (indexing was carried out with the IDT for Illumina DNA/RNA UD Indexes Set A) according to the manufacturer’s protocol. Equimolar concentrations of all samples were pooled and sequenced on an Illumina MiSeq instrument (Illumina, San Diego, CA, USA) using the paired-end (2 × 300 bp) method with the MiSeq-v3 reagent kit (Illumina, San Diego, CA, USA). The read quality of the generated files was determined using FastQC, and the low-quality sequences were trimmed using Trimmomatic. The resultant reads were aligned/mapped to HIV-1 reference (RefSeq: NC_001802.1) using the BWA to generate viral contigs. HIV-1 subtyping was performed using the REGA-v3 HIV-1 Subtyping Tool, [76] and any refractory sequences/samples were resolved using the RIP tool (https://www.hiv.lanl.gov/content/sequence/RIP/RIP.html, accessed on 24 March 2023) or HIV-1 blast (https://www.hiv.lanl.gov/content/sequence/BASIC_BLAST/basic_blast.html, accessed on 24 March 2023) followed by phylogenetic analysis with PhyML (https://www.hiv.lanl.gov/content/sequence/PHYML/interface.html, accessed on 24 March 2023).

### 4.8. HIV-1-Specific IFN-γ and IL-2 Dual ELISpot Assay

HIV-1-specific HLA-C*03:02-restricted CD8^+^ T cell responses were evaluated using a dual ELISpot assay. HIV-1 peptides were synthesized using the Fmoc (fluoren-9-ylmethoxycarbonyl) means of solid–phase peptide synthesis technology and the purity was confirmed using high-pressure liquid chromatography (Bio-Synthesis, Inc Lewisville, TX). Peptides were diluted to a final concentration of 10 µg/mL. PBMCs were isolated by density gradient centrifugation from EDTA whole blood and cryopreserved. Frozen PBMCs were thawed, and viability was confirmed by trypan blue, then rested overnight before plating. We evaluated the secretion of IFN-γ and/or IL-2 by PBMC using the ELSP5710/5810 AID iSpot FluoroSpot kit (AID Autoimmun Diagnostika GmbH, Straßberg, Germany) according to the manufacturer’s instructions. Briefly, 96-well plates pre-coated with both IFN-γ and IL-2 monoclonal antibodies were incubated with 100 µL of 2 × 10^5^ viable cells and 100 µL of peptide solution per well at 37 °C in humidified 5% CO_2_ for 40 h. Media alone was used as a negative control (NC), and pokeweed as a positive control. Plates were washed and stained with biotinylated anti-human IL-2 and anti-human IFN-γ FITC. IFN-γ and IL-2 production was quantified using an AID iSpot EliSpot/FluoroSpot Reader (AID Autoimmun Diagnostika GmbH, Straßberg, Germany) and expressed as spot-forming cells (SFC) per million PBMC after subtraction of background spots from NC. A Fisher’s exact test was used for comparing proportions of ELISPOT responses in the HLA-C*03:02^+ve^ and C*03:02^−ve^ participants.

## 5. Conclusions

In conclusion, we have used an immunoinformatics approach to identify a potentially HLA-C*03:02-restricted epitope, eliciting T cell-specific responses, suggesting that the GY9 epitope may play a significant role in HLA-C*03:02-mediated HIV-1 control among children. This study provides additional support for the hypothesis that an effective HIV-1 vaccine could be clade-specific; therefore, efforts for a global vaccine may not be feasible. And as such, more focus may be placed on identifying possible epitopes mapped across all clades, including those restricted to protective HLA-C alleles. Finally, our study expands upon prior studies by providing evidence supporting the notion that the HIV-1 matrix protein p17 represents a promising epitope candidate for developing a vaccine against HIV/AIDS.

## Figures and Tables

**Table 1:** Molecular docking results of top-ranked HIV epitopes docked with C*0302.

HIV-1 Clade	Protein and Position	Peptide Sequence		No. of H Bonds	RMSD ^a^	Binding Energy (kcal/mol)	Convex-PL Score	Amino Acids Involved in H Bond Interaction
	**Structural**							
C/A1	Gag^71^ *	GTEELRSLY	GY9	7	0.811	–8.6	7.23	Lys65, Tyr83, Tyr98, Thr142, Trp146 and Tyr158
A1	Env^58^	KAYETEMHN	KN9	11	0.202	–7.4	7.36	Tyr6, Arg61, Glu62, Lys65, Tyr66, Ser76, Glu151 and Tyr158
C	Pol^43^ *	GAERQGTLNF	GF10	7	1.165	–8.7	7.22	Tyr6, Glu62, Lys65, Tyr98, Thr142, Lys145 and Gln154
C	Pol^324^ *	AQNPEIVIY	AY9	6	1.547	–7.7	7.16	Glu62, Lys65, Lys145, Tyr158 and Tyr170
	**Regulatory**							
C/A1	Nef^84^	GAFDLSFFL	GL9	7	1.030	–9.1	7.24	Tyr8, Glu62, Lys65, Thr72, Tyr98, Lys145 and Tyr158
C/A1	Nef^114^	WVYNTQGYF	WF9	7	0.873	–9.7	7.53	Tyr6, Arg68, Tyr98, Thr142, Lys145, Trp146 and Glu151
A1	Vif^128^	VVSPRCEY	VY8	8	1.473	–8.3	7.10	Gln69, Thr72, Ser76, Tyr83, Tyr98, Thr142, Ly145 and Glu151
A1	Rev^109^ *	VSVESPVIL	VL9	7	1.638	–8.0	7.27	Tyr8, Arg61, Thr72, Asn79, Tyr83 and Tyr98

^a^ RMSD: root mean square deviation (in Å) in comparison to the native ligand (PDB ID: 5w6a.2) of the C*0602 allele. * stable on molecular dynamics.

**Table 2:** Binding free energies obtained by the MM/PBSA method of pHLA complexes with GY9, GF10, AY9, and VL9 peptide.

HIV-1 Subtype	Peptide Sequence		Energy Components (kcal/mol)			
			Van der Waals	Electrostatics	Polar Solvation	ΔG Binding Energy
	**Structural**					
C/A1	GTEELRSLY	GY9	−53.01	−547.44	521.66	−88.41
C	GAERQGTLNF	GF10	−53.45	−335.96	347.44	−50.59
C	AQNPEIVIY	AY9	−68.75	−258.85	285.79	−51.24
	**Regulatory**					
A1	VSVESPVIL	VL9	−67.29	−367.42	394.36	−49.73

**Table 3:** Change in binding free energy and conservancy scores of the GY9 peptide.

HIV1 Clade ^a^	Amino Acid Residue and Position								
	G1	T2	E3	E4	L5	R6	S7	L8	Y9
A1	.	.	.	.	.	R/K	.	.	Y/F
C	.	.	.	.	.	K	.	.	Y/F/H
D	.	.	.	.	I	K	.	.	Y/F
K	.	.	.	.	I	K	.	.	Y/F
ΔΔG GY9 ^b^	NA	−8.18	−10.3	−4.82	−2.83	−27.00	−5.85	−2.82	−7.33
Conservancy score ^c^	5	4	2	3	3	2	5	5	2

^a^ Common clades in Botswana and Uganda’s populations; NA, glycine is of similar size to alanine. ^b^ Binding free energy change due to mutation of amino acid residue to alanine. ^c^ The scores are from one to nine to show the conservation level (low to high, respectively).

## Data Availability

Data are contained within the article and supplementary materials.

## Supplementary Materials

The following supporting information can be downloaded at: https://www.mdpi.com/article/10.3390/ijms25179683/s1.
