# Supplementary material for: Identification of a Clade-Specific HLA-C*03:02 CTL Epitope GY9 Derived from the HIV-1 p17 Matrix Protein
Source: Int J Mol Sci. 2024 Sep 6;25(17):9683. doi: 10.3390/ijms25179683 (PMC11395705; doi:10.3390/ijms25179683)
Supplement: Supplementary file 1 [file ijms-25-09683-s001.zip › ijms-2677517-supplementary.pdf]

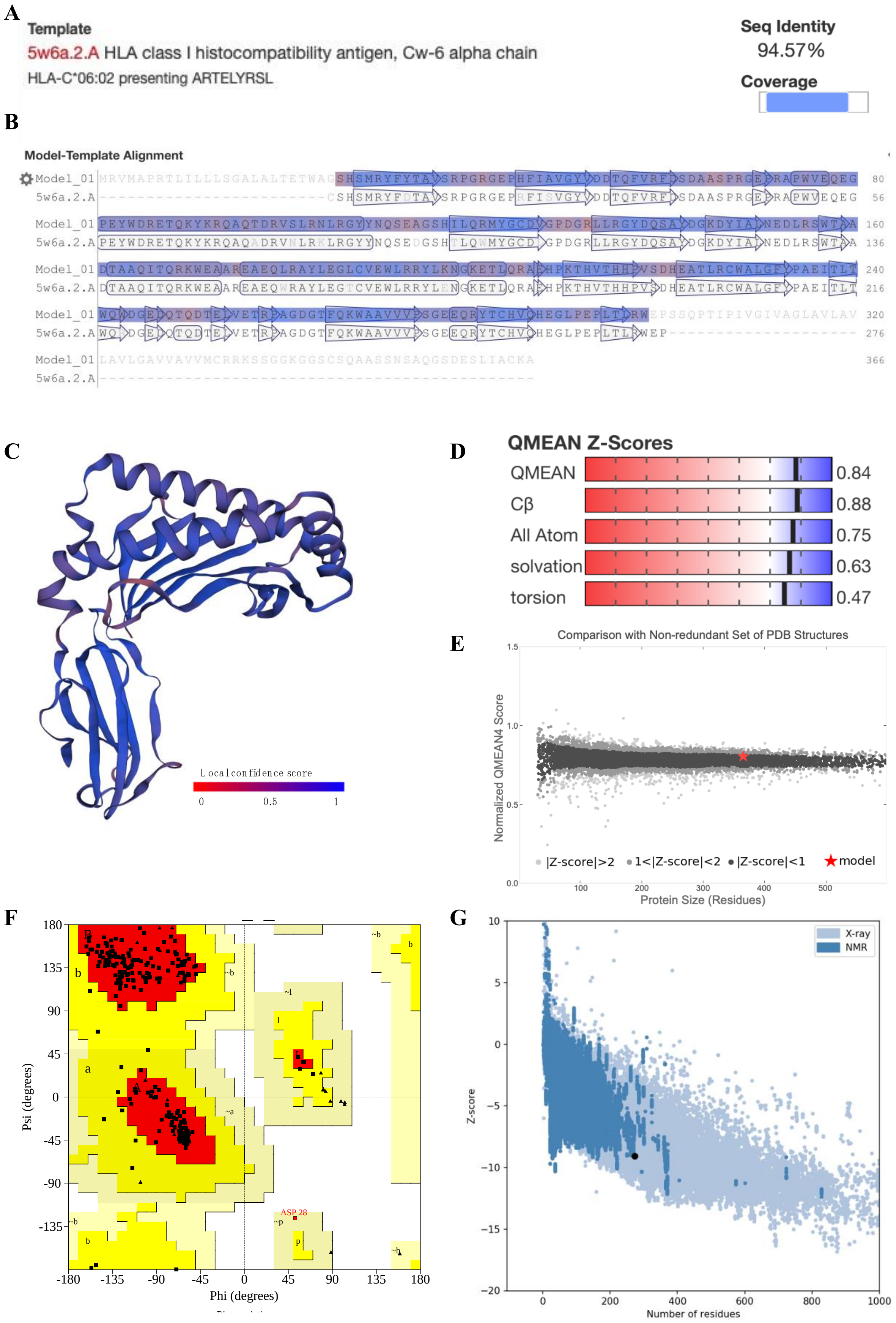

Figure S1. Model building and validation. (A) Parameters of 5w6a.2.A the template selected for model building. (B) Model-template alignment. The predicted model is colored by a QMEANDisCo score similar to Figure 1C. (C) 3D structure of the model colored by local residue confidence score, red being low and blue very high score estimated with the QMEANDisCo scoring function (range 0–1). An oval ring shows the peptide binding groove. (D) QMEAN z-scores of the model. All five scores are a comparison of the model with the expected experimentally determined structures of similar size.

(E) Plot of the normalized QMEAN scores of the reference set used in constructing the protein model of C*0302. (F) Ramachandran plot shows the values of dihedral angles Φ and Ψ of each residue. Red indicates favored region, brown shows the allowed region, while yellow and light yellow are outlier regions. No residue lies outside of the favored region. (G) ProSA – Protein structural analysis plot of the overall 3D quality z-score of the model compared to X-ray and NMR solved structures.

Abbreviations: QMEAN, Qualitative Model Energy Analysis; QMEANDisCo, Qualitative Model Energy Analysis Distance constraints.

A

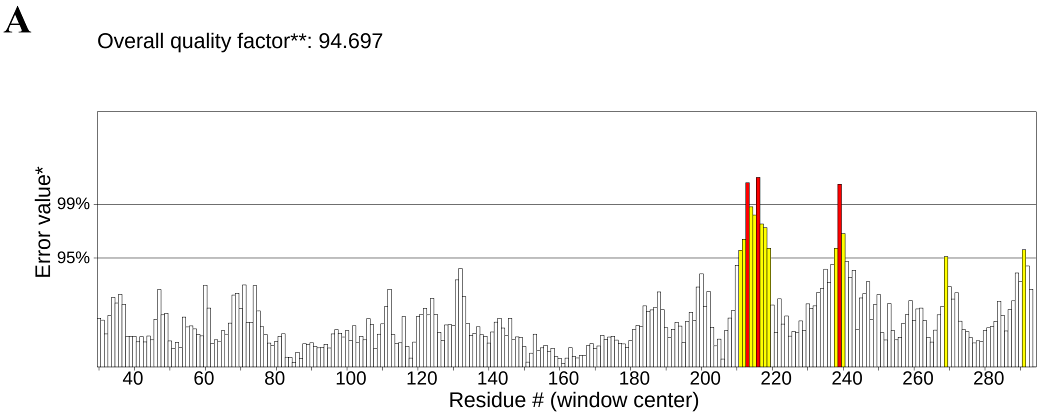

B

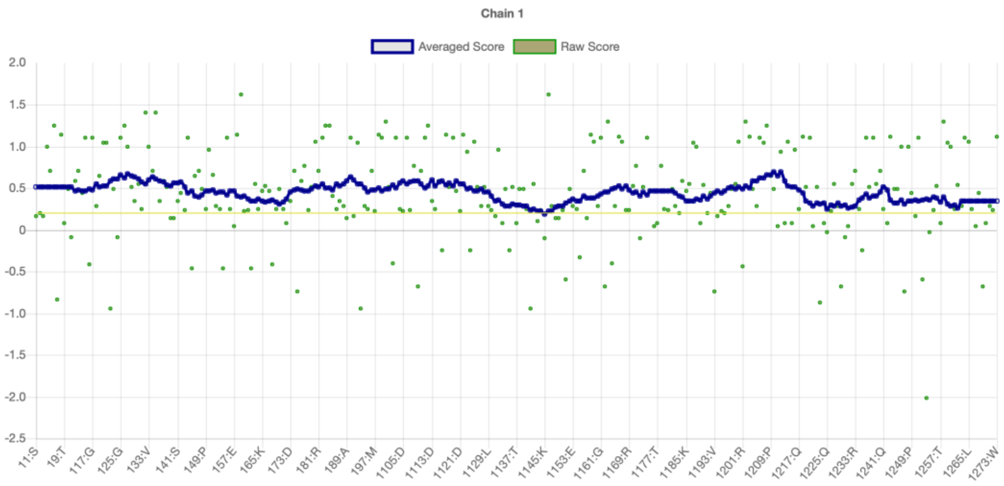

C

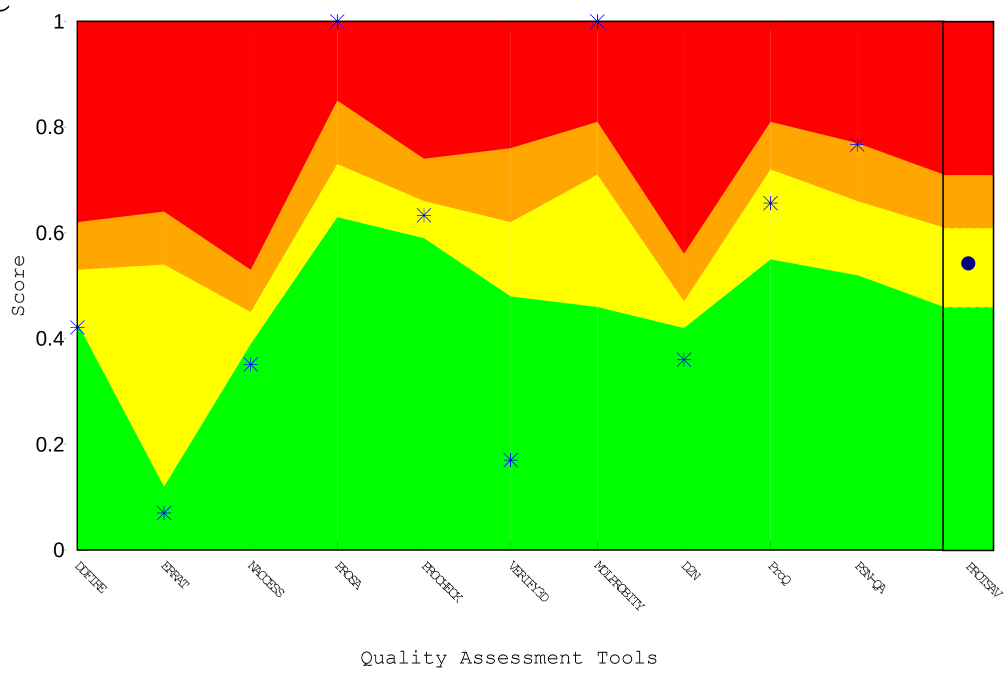

Figure S2. Modeling validation. (A) ERRAT score for the selected model. Yellow color shows the error region between 95 and 99%, Red color shows the misfolded region, and white color shows the lower error rate of protein folding. (B) Verify-3D plot showed that 99.63% of the residues have averaged a 3D-1D score of ≥ 0.2. (C) ProTSAV score of 0.54 and root-mean-square distance (RMSD) within the range of 2-5Å, suggesting moderate accuracy of the model.

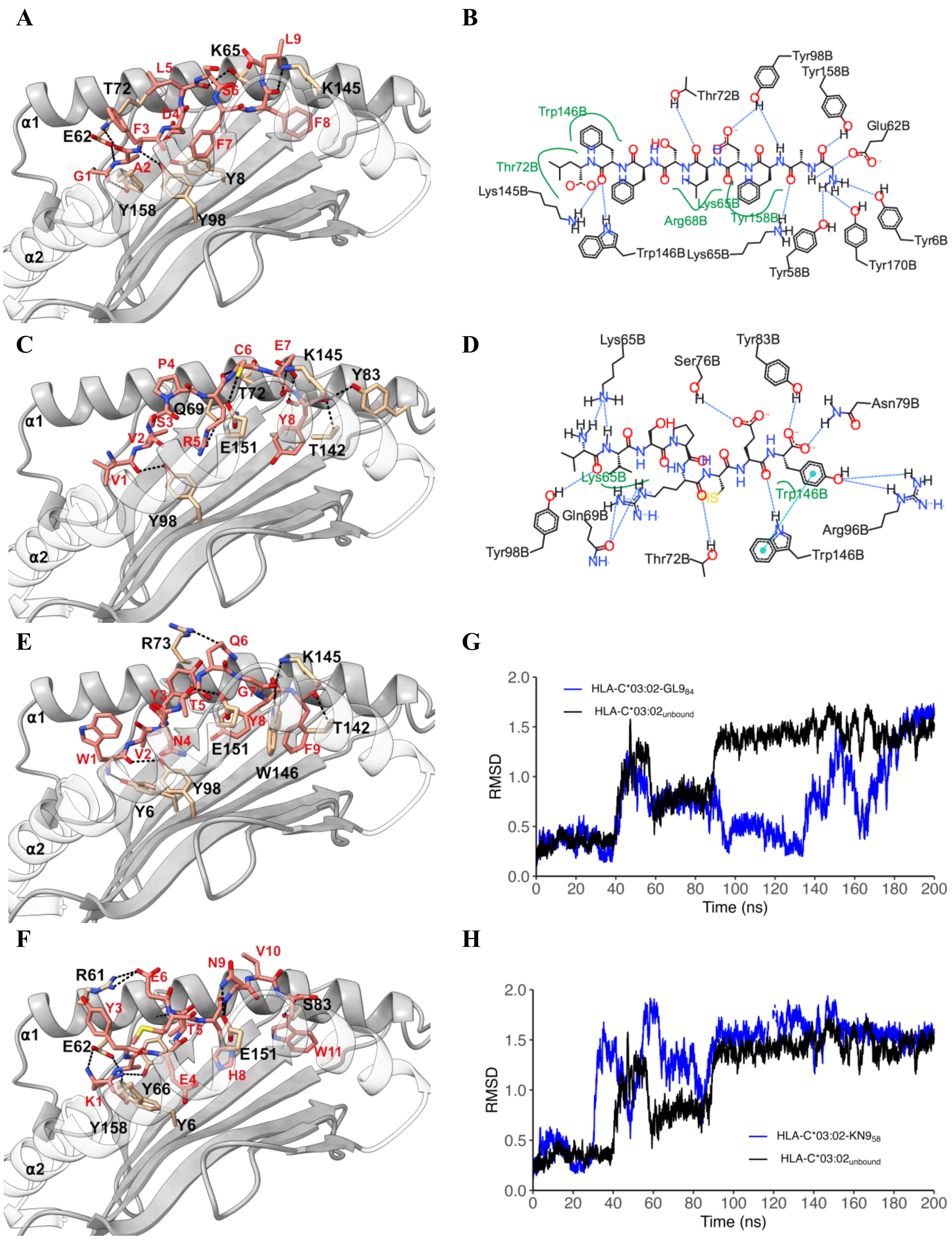

Figure S3. The molecular docking representation and RMSD analysis of C*0302 with docked epitopes GL9 (A, B and G), VY8 (C&D), WF9 (E) and KN9 (F and H). (G) and (H) show the RMSD plot of GL9 and KN9 showing the lack of stability.

Table S1. List of peptides classified as strong or weak binders

Peptide	Protein	Position	Subtype	Score_EL	%Rank_EL	Score_BA	%Rank_BA	Aff(nM)	Shared with MotifScan	Binding Level
RAVGIGAVF	Env	493/4	A1 & C	0.882943	0.029	0.830774	0.023	6.24	Y	SB
RAVGIGAVF	Env	494	C	0.882943	0.029	0.830774	0.023	6.24		SB
ISNYTHIIY	Env	617	A1	0.761367	0.089	0.757922	0.072	13.73		SB
LAWDDLRSL	Env	737	A1 & C	0.742696	0.098	0.694673	0.166	27.21		SB
RAIEAQQHL	Env	539	A1	0.719846	0.124	0.621234	0.337	60.23		SB
FSYHRLRDF	Env	748	A1 & C	0.715692	0.127	0.785577	0.048	10.18		SB
VSFEPIPIHY	Env	203	A1	0.572694	0.24	0.513237	0.791	193.77		SB
HSFNCGGEF	Env	367	A1	0.531917	0.286	0.765019	0.064	12.71		SB
FCASDAKAY	Env	52	A1 & C	0.47309	0.366	0.582521	0.478	91.56		SB
NAKTIIVQL	Env	275	A1	0.451979	0.395	0.355564	2.382	1067.05		SB
SFEPIPIHY	Env	204	A1	0.436763	0.418	0.251438	4.882	3292.08		SB
SAAENLWVTVY	Env	28	A1	0.398212	0.478	0.507083	0.826	207.11	y	SB
RAIEAQQHM	Env	529	C	0.83873	0.05	0.72826	0.116	18.92		SB
VSFDPIPIHY	Env	196	C	0.67235	0.159	0.526122	0.725	168.55		SB
TSKLFNSTY	Env	373	C	0.660196	0.172	0.607834	0.378	69.63		SB
SFDPIPIHY	Env	197	C	0.653036	0.178	0.296926	3.526	2012.44		SB
YSPLSFQTL	Env	684	C	0.523339	0.297	0.592497	0.435	82.19		SB
TAVPWNSSW	Env	578	C	0.498843	0.33	0.553949	0.6	124.73		SB
ISNYTDTIY	Env	607	C	0.49404	0.337	0.680644	0.192	31.67		SB
HSFNCRGEF	Env	360	C	0.447834	0.4	0.722646	0.124	20.1		SB
NATNATNTM	Env	132	C	0.434379	0.422	0.622937	0.332	59.13		SB
YTDTIYRLL	Env	610	C	0.428991	0.43	0.517677	0.768	184.68		SB
SIRIGPGQTF	Env	294	C	0.395192	0.483	0.43147	1.402	469.36		SB
VGNLWVTVY	Env	30	C	0.386815	0.496	0.521671	0.748	176.87		SB

RALGPGATL	Gag	335	A1 & C	0.769621	0.085	0.698606	0.157	26.08		SB
FSPEVIPMF	Gag	164	A1 & C	0.601813	0.216	0.573088	0.519	101.4		SB
YVDRFFKTL	Gag	296	A1 & C	0.512706	0.311	0.532807	0.692	156.79		SB
NSSKVSQNY	Gag	124	A1	0.463877	0.378	0.471721	1.06	303.65		SB
FALNPSLL	Gag	44	A1	0.414924	0.452	0.532447	0.694	157.4		SB
IALEMHPEF	Nef	195	A	0.911434	0.016	0.779453	0.051	10.87		SB
YTPGPGTRF	Nef	128	A	0.731763	0.109	0.550209	0.616	129.88		SB
MARELHPEY	Nef	195	C	0.927552	0.013	0.802619	0.041	8.46		SB
YTPGPGVRY	Nef	128	C	0.799714	0.067	0.562132	0.565	114.16		SB
AAFDLSFFL	Nef	84	C	0.447516	0.401	0.601996	0.396	74.17		SB
QVPLRPMTY	Nef	74	C	0.422209	0.441	0.346374	2.524	1178.61		SB
KAAFDLSFF	Nef	83	C	0.401476	0.473	0.634637	0.297	52.1		SB
FSVPLDESF	Pol	271	A1	0.801681	0.066	0.774497	0.055	11.47		SB
VAVHVASGY	Pol	790	A1 & C	0.734988	0.105	0.733173	0.109	17.94		SB
FSFPQITL	Pol	54	A1	0.709584	0.131	0.611932	0.366	66.61		SB
FSFPQITLW	Pol	54	A1	0.611108	0.209	0.624421	0.328	58.19		SB
KAQEEHERY	Pol	722	A1	0.567706	0.244	0.422455	1.503	517.45		SB
TVLDVGDAY	Pol	262	A1	0.508985	0.316	0.536598	0.676	150.49		SB
WANIQQEF	Pol	847	A1	0.456545	0.388	0.488867	0.943	252.23		SB
KALTDIVTL	Pol	442	A1	0.434042	0.422	0.48895	0.942	252		SB
IQQEFGIPY	Pol	850	A1 & C	0.422751	0.44	0.561249	0.569	115.26		SB
IVTDSQYAL	Pol	650	A1 & C	0.422403	0.44	0.525228	0.73	170.19		SB
VIWGKTPKF	Pol	536	A1 & C	0.417946	0.447	0.396999	1.785	681.53		SB
YAGIKVKQL	Pol	426	A1	0.416351	0.45	0.433945	1.379	456.96		SB
FQQGEARKF	Pol	8	A1	0.399945	0.476	0.413004	1.597	573.16		SB
IIRDYGKQM	Pol	982	A1	0.385613	0.498	0.447044	1.256	396.57		SB

AQNPEIVIY	Pol	324	C	0.633581	0.192	0.405893	1.675	619		SB
KAQEEHEKY	Pol	718	C	0.629062	0.195	0.416035	1.567	554.67		SB
LAFPQGEAREF	Pol	6	C	0.503965	0.323	0.55705	0.587	120.62		SB
IAMESIVIW	Pol	526	C	0.453247	0.393	0.584738	0.469	89.39		SB
FSVPLDEGF	Pol	267	C	0.420922	0.443	0.643744	0.274	47.21		SB
IIKDYGKQM	Pol	978	C	0.411548	0.457	0.411849	1.609	580.37		SB
RAQNPEIVIY	Pol	323	C	0.395124	0.483	0.440535	1.319	425.51		SB
SAEPVPLQL	Rev	67	A1	0.826617	0.055	0.504657	0.841	212.62		SB
VSVESPVIL	Rev	109	A1	0.560202	0.25	0.582496	0.478	91.59		SB
QTKGLGISY	Tat	39	C	0.5222	0.298	0.410085	1.629	591.55		SB
VSSEVHIPL	Vif	51	A1 & C	0.527779	0.291	0.697677	0.159	26.34		SB
QVVSPRCEY	Vif	127	A1	0.517982	0.304	0.510408	0.806	199.79		SB
FPRPWLHSL	Vpr	34	C	0.39766	0.479	0.453593	1.193	369.45		SB
SIRIGPGQAF	Env	301	A1	0.382582	0.504	0.475587	1.035	291.21		WB
IAARTVEL	Env	759	A1	0.366525	0.54	0.401091	1.732	652.01		WB
KSIRIGPGQAF	Env	300	A1	0.355927	0.564	0.431358	1.404	469.93		WB
VAKQLRKYF	Env	339	A1	0.353734	0.569	0.399043	1.759	666.62	Y	WB
VSFEPIPIH	Env	203	A1	0.307562	0.672	0.387403	1.905	756.09		WB
KVAKQLRKY	Env	338	A1	0.294791	0.701	0.309951	3.231	1747.91		WB
IAARTVELL	Env	759	A1	0.291271	0.711	0.512399	0.795	195.53		WB
LIAARTVEL	Env	758	A1	0.284241	0.733	0.528858	0.711	163.64		WB
YAPPIQGVI	Env	418	A1	0.270802	0.774	0.515124	0.781	189.85		WB
LALDKWANL	Env	643	A1	0.267282	0.785	0.491717	0.922	244.57		WB
KRAVGIGAVF	Env	492	A1 & C	0.250841	0.84	0.56029	0.573	116.46		WB
FLGAAGSTM	Env	504	A1 & C	0.248595	0.847	0.637507	0.29	50.51		WB
HSSLKGLRL	Env	769	A1	0.235975	0.891	0.385263	1.937	773.8		WB

TQACPKVSF	Env	197	A1 & C	0.230732	0.911	0.381144	1.998	809.07		WB
TVYYGVPVW	Env	36	A1 & C	0.230436	0.912	0.448681	1.24	389.61		WB
LTVQARQLL	Env	519	A1 & C	0.229157	0.918	0.483657	0.98	266.86		WB
CSFNMTTEL	Env	150	A1	0.227933	0.923	0.658451	0.238	40.27		WB
GTMKNTITL	Env	391	A1	0.223283	0.943	0.456227	1.173	359.06		WB
IRIGPGQAF	Env	302	A1	0.221586	0.95	0.394757	1.813	698.26		WB
RIGPGQAFY	Env	303	A1	0.22056	0.954	0.363576	2.262	978.45		WB
YFKNKTIIF	Env	346	A1	0.215331	0.976	0.371941	2.136	893.78		WB
RSIRLVSGF	Env	726	A1 & C	0.203926	1.032	0.527622	0.717	165.84		WB
LTVWGIKQL	Env	550	A1 & C	0.187713	1.125	0.408074	1.651	604.56		WB
RLVSGFLAL	Env	729	A1 & C	0.180556	1.172	0.509643	0.811	201.45		WB
YSPLSFQTH	Env	694	A1	0.179217	1.18	0.403213	1.705	637.21		WB
EAQQHLLKL	Env	542	A1	0.178821	1.183	0.262888	4.51	2908.49		WB
YCAPAGFAI	Env	212	A1	0.17839	1.186	0.513188	0.791	193.87		WB
FTNSSGGDL	Env	354	A1	0.177427	1.192	0.581559	0.482	92.52		WB
VINRVRQGY	Env	686	A1	0.173406	1.219	0.333906	2.752	1348.83		WB
GAASITLTV	Env	513	A1 & C	0.153775	1.361	0.442535	1.301	416.4		WB
WQRAGQAMY	Env	410	A1	0.141279	1.472	0.44034	1.321	426.41		WB
RQGYSPLSF	Env	691	A1 & C	0.13327	1.546	0.335467	2.723	1326.24		WB
IVQQQSNLL	Env	530	A1 & C	0.127759	1.598	0.308892	3.255	1768.05		WB
RIGPGQAF	Env	303	A1	0.127464	1.601	0.261377	4.559	2956.43		WB
RVIEIGQRI	Env	817	A1	0.126817	1.608	0.325536	2.905	1476.68		WB
SAAENLWVTVYY	Env	28	A1	0.123482	1.643	0.43549	1.365	449.38		WB
VTNNTTNTH	Env	135	A1	0.122096	1.658	0.32029	3	1562.92		WB
HLENVTEEF	Env	84	A1	0.119104	1.69	0.281899	3.926	2367.75		WB
RVRQGYSPL	Env	689	A1 & C	0.118692	1.694	0.498367	0.879	227.59		WB

KVSFEPIPIHY	Env	202	A1	0.116024	1.728	0.201961	7.138	5622.91		WB
IISLWDQSL	Env	107	A1 & C	0.110283	1.804	0.425397	1.471	501.24		WB
QGYSPLSF	Env	692	A1 & C	0.105649	1.866	0.22955	5.738	4171.78		WB
VAKQLRKY	Env	339	A1	0.105324	1.87	0.160003	10.071	8853.62	Y	WB
HSFNCGGEFF	Env	367	A1	0.104954	1.875	0.508729	0.817	203.45		WB
KQKVYSLFY	Env	162	A1	0.104182	1.885	0.31117	3.204	1725.01		WB
QACPKVSF	Env	198	A1 & C	0.101887	1.916	0.172265	9.063	7753.57	Y	WB
QMHTDIISL	Env	102	A1	0.099229	1.953	0.342537	2.594	1228.57		WB
CASDAKAY	Env	53	A1 & C	0.09624	1.995	0.308427	3.265	1776.97	Y	WB
MIVGGLIGL	Env	669	A1 & C	0.09606	1.998	0.437634	1.346	439.08		WB
VSFDPIPIH	Env	196	C	0.34104	0.598	0.393241	1.832	709.81		WB
CSFNITTEL	Env	146	C	0.337204	0.607	0.663407	0.227	38.16		WB
KSIRIGPGQTF	Env	293	C	0.335109	0.611	0.377459	2.053	841.98		WB
IVNRVRQGY	Env	676	C	0.319602	0.646	0.423979	1.486	508.99		WB
NAKTIIVHL	Env	268	C	0.318164	0.649	0.323603	2.94	1507.89		WB
RIGPGQTFY	Env	296	C	0.293707	0.704	0.347757	2.499	1161.1		WB
IAARAVEL	Env	749	C	0.281786	0.74	0.358909	2.332	1029.12		WB
EAQQHMLQL	Env	532	C	0.267757	0.783	0.335862	2.716	1320.58		WB
LALDSWKNL	Env	633	C	0.263671	0.796	0.464013	1.113	330.06		WB
IRIGPGQTF	Env	295	C	0.253702	0.83	0.361972	2.286	995.58		WB
NSTNSTITL	Env	382	C	0.25187	0.836	0.475012	1.039	293.02		WB
IAARAVELL	Env	749	C	0.24898	0.846	0.509625	0.811	201.49		WB
QMHEDIISL	Env	102	C	0.226914	0.927	0.402093	1.719	644.98		WB
FDPIPIHY	Env	198	C	0.215602	0.975	0.167134	9.491	8196.2		WB
YAILKCNNKTF	Env	211	C	0.203654	1.033	0.536423	0.676	150.78		WB
LVQYWGLEL	Env	778	C	0.195315	1.08	0.552698	0.605	126.43		WB

LIAARAVEL	Env	748	C	0.192955	1.093	0.475596	1.035	291.18		WB
YCAPAGYAI	Env	205	C	0.190789	1.105	0.509632	0.811	201.48		WB
KVSFDPIPIHY	Env	195	C	0.174861	1.209	0.21699	6.32	4779.04		WB
KSNITGLLL	Env	420	C	0.148587	1.404	0.420552	1.522	528.21		WB
YAPPIAGNI	Env	409	C	0.145736	1.43	0.428847	1.432	482.87		WB
FFYCNTSKL	Env	368	C	0.13947	1.489	0.543822	0.644	139.18		WB
YTDTIYRL	Env	610	C	0.132907	1.55	0.242296	5.213	3634.36		WB
LRAIEAQQHM	Env	528	C	0.130522	1.572	0.447056	1.256	396.52		WB
RIGPGQTF	Env	296	C	0.127788	1.598	0.232341	5.613	4047.68		WB
KYLGSLVQY	Env	773	C	0.126143	1.615	0.208671	6.756	5229.15		WB
IVLENVTENF	Env	83	C	0.119627	1.684	0.371536	2.142	897.7		WB
VSKKLKEHF	Env	332	C	0.11672	1.719	0.21887	6.23	4682.81		WB
ILKCNNKTF	Env	213	C	0.107861	1.836	0.362187	2.283	993.26		WB
FPNKTIKF	Env	340	C	0.104282	1.884	0.185568	8.115	6714.16		WB
MWQEVGRAM	Env	400	C	0.103597	1.893	0.384677	1.945	778.73		WB
STNSTITL	Env	383	C	0.10245	1.909	0.23879	5.349	3774.88		WB
YLGSLVQY	Env	774	C	0.097504	1.978	0.237775	5.388	3816.56		WB
SQVQHTNIM	Gag	368	A1	0.335296	0.611	0.540339	0.659	144.52		WB
IVGGHQAAM	Gag	190	A1	0.332089	0.618	0.531842	0.696	158.44		WB
RMYSPVSIL	Gag	275	A1 & C	0.300269	0.688	0.568383	0.539	106.7		WB
HQSLSPRTL	Gag	144	A1	0.27481	0.762	0.343764	2.572	1212.36		WB
EQDPPLVSL	Gag	479	A1	0.265043	0.792	0.168239	9.399	8098.79		WB
EVIPMFSAL	Gag	167	A1	0.230496	0.912	0.448962	1.237	388.43		WB
KARVLAEAM	Gag	359	A1 & C	0.22823	0.922	0.534478	0.685	153.98		WB
SLYNTVATL	Gag	77	A1 & C	0.227893	0.923	0.477865	1.02	284.12		WB
SSKGRPGNF	Gag	438	A1	0.207597	1.012	0.332815	2.772	1364.84		WB

WASRELERF	Gag	36	A1 & C	0.196382	1.074	0.457215	1.165	355.25		WB
HLVWASREL	Gag	33	A1 & C	0.184639	1.145	0.46826	1.083	315.23		WB
FSPEVIPM	Gag	164	A1 & C	0.184534	1.146	0.511259	0.801	197.96		WB
GATPQDLNM	Gag	178	A1	0.178777	1.183	0.354918	2.392	1074.54		WB
LRALGPGATL	Gag	334	A1 & C	0.176845	1.196	0.468908	1.079	313.03		WB
KAFSPEVIPM	Gag	162	A1 & C	0.162729	1.291	0.570562	0.529	104.21		WB
LYNTVATLY	Gag	78	A1 & C	0.158278	1.325	0.341834	2.607	1237.95		WB
GTEELRSLY	Gag	71	A1 & C	0.157273	1.333	0.290496	3.697	2157.44		WB
KAFSPEVIPMF	Gag	162	A1 & C	0.138827	1.495	0.325694	2.902	1474.16		WB
IVQNAQGQM	Gag	134	A1	0.136572	1.516	0.386398	1.92	764.36		WB
SSKVSQNY	Gag	125	A1	0.130482	1.572	0.205323	6.943	5422.05		WB
ILRALGPGATL	Gag	333	A1 & C	0.127028	1.605	0.327163	2.875	1450.91		WB
HAGPIPPGQM	Gag	219	A1	0.119532	1.685	0.264835	4.449	2847.86		WB
KVIEEKAF	Gag	157	A1 & C	0.105035	1.874	0.190356	7.822	6375.19		WB
AFSPEVIPM	Gag	163	A1 & C	0.097858	1.973	0.318572	3.039	1592.24		WB
RAEQATQEV	Gag	305	A1	0.097632	1.976	0.33118	2.802	1389.2		WB
KAADGKVSQNY	Gag	119	C	0.381886	0.506	0.328432	2.852	1431.13		WB
FALNPGLL	Gag	44	C	0.327012	0.629	0.500559	0.866	222.26		WB
QANNTNIMM	Gag	366	C	0.316923	0.651	0.534963	0.683	153.18		WB
QAISPRTL	Gag	142	C	0.301281	0.686	0.286115	3.814	2262.16		WB
HQAISPRTL	Gag	141	C	0.299385	0.69	0.376814	2.063	847.88		WB
EVIPMFTAL	Gag	164	C	0.268549	0.781	0.434537	1.374	454.04		WB
AADGKVSQNY	Gag	120	C	0.246056	0.856	0.269077	4.315	2720.1		WB
TAPPAESF	Gag	452	C	0.210741	0.996	0.249181	4.956	3373.46		WB
TVGGHQAAM	Gag	187	C	0.208381	1.007	0.434397	1.375	454.73		WB
IIKQLQPAL	Gag	60	C	0.177103	1.194	0.335437	2.724	1326.67		WB

IAGTTSTL	Gag	233	C	0.147722	1.412	0.319726	3.013	1572.49		WB
SQANNTNIM	Gag	365	C	0.139661	1.487	0.463489	1.117	331.93		WB
IVQNLQGQM	Gag	131	C	0.119719	1.683	0.353613	2.411	1089.82		WB
ATPQDLNTM	Gag	176	C	0.116598	1.721	0.283877	3.873	2317.61		WB
MTSNPPIPV	Gag	247	C	0.115201	1.739	0.549135	0.621	131.4		WB
PTAPPAESF	Gag	451	C	0.113002	1.768	0.21742	6.3	4756.86		WB
WVYNTQGYF	Nef	114	A	0.357859	0.56	0.68489	0.184	30.25		WB
IALEMHPEFY	Nef	195	A	0.339334	0.602	0.512904	0.793	194.47		WB
QVPLRPMTF	Nef	74	A	0.301437	0.686	0.316784	3.079	1623.35		WB
YSQKRQEIL	Nef	103	A	0.241465	0.872	0.446334	1.263	399.63		WB
GAFDLSFFL	Nef	84	A	0.228536	0.92	0.491318	0.925	245.63		WB
HIALEMHPEF	Nef	194	A	0.16498	1.275	0.460682	1.139	342.17		WB
MTFKGAFDL	Nef	80	A	0.143811	1.448	0.586978	0.459	87.25		WB
WVYNTQGY	Nef	114	A	0.133409	1.545	0.385191	1.938	774.41		WB
FPVRPQVPL	Nef	69	A & C	0.120275	1.677	0.441135	1.314	422.76		WB
GAFDLSFF	Nef	84	A	0.117278	1.712	0.318159	3.048	1599.38		WB
AATQASCAW	Nef	49	A	0.109135	1.82	0.431006	1.408	471.72		WB
LARRHIAL	Nef	190	A	0.09746	1.978	0.326682	2.884	1458.48		WB
MARELHPEYY	Nef	195	C	0.361584	0.552	0.590551	0.444	83.94		WB
WVYHTQGYF	Nef	114	C	0.28466	0.732	0.642211	0.277	48		WB
AAFDLSFF	Nef	84	C	0.28226	0.739	0.421807	1.51	521.09		WB
HMARELHPEY	Nef	194	C	0.26171	0.802	0.467187	1.09	318.91		WB
YSKKRQEIL	Nef	103	C	0.209644	1	0.380848	2.003	811.66		WB
YKAAFDLSF	Nef	82	C	0.199563	1.056	0.571632	0.525	103.01		WB
KAAFDLSF	Nef	83	C	0.176032	1.201	0.398621	1.764	669.67		WB
WVYHTQGY	Nef	114	C	0.164097	1.281	0.372584	2.127	887.58		WB

VSSGIRKVL	Pol	707	A1 & C	0.36556	0.543	0.451144	1.215	379.37		WB
ESFRKYTAF	Pol	277	A1	0.3559	0.564	0.510061	0.809	200.54		WB
MAVFIHNF	Pol	893	A1 & C	0.312	0.662	0.497293	0.885	230.25		WB
KTAVQMAVF	Pol	888	A1 & C	0.286168	0.727	0.557407	0.585	120.15		WB
SKNPEIIIY	Pol	328	A1	0.282558	0.738	0.27689	4.07	2499.61		WB
LKDPVHGVY	Pol	465	A1	0.275662	0.759	0.371671	2.14	896.39		WB
KIQNFRVYY	Pol	934	A1 & C	0.26776	0.783	0.401027	1.733	652.46		WB
QSQGVVESM	Pol	861	A1 & C	0.252004	0.836	0.412605	1.601	575.64		WB
FVNTPPLVKL	Pol	571	A1 & C	0.239215	0.88	0.459389	1.149	346.99		WB
KQITKIQNF	Pol	930	A1	0.224701	0.937	0.303688	3.371	1870.46		WB
LTEEKIKAL	Pol	181	A1 & C	0.224105	0.939	0.326478	2.887	1461.7		WB
ILKDPVHGVY	Pol	464	A1	0.223912	0.94	0.352937	2.421	1097.82		WB
FQSSMTKIL	Pol	315	A1 & C	0.218146	0.964	0.504703	0.841	212.51		WB
TAVQMAVF	Pol	889	A1 & C	0.206477	1.018	0.431294	1.404	470.25		WB
KLNWASQIY	Pol	418	A1 & C	0.204547	1.028	0.449963	1.227	384.24		WB
LGIPHPAGL	Pol	247	A1 & C	0.200432	1.051	0.413018	1.597	573.07		WB
QGTGPTFSF	Pol	48	A1	0.192154	1.097	0.329447	2.833	1415.49		WB
VVMESIVIW	Pol	530	A1	0.19088	1.105	0.357055	2.36	1049.98		WB
TKIQNFRVY	Pol	933	A1	0.188567	1.12	0.383669	1.96	787.26		WB
ISKIGPENPY	Pol	202	A1	0.187844	1.124	0.489952	0.935	249.29		WB
KLIGKDKVY	Pol	679	A1	0.1755	1.205	0.29419	3.599	2072.91		WB
RAHLLSWGF	Pol	361	A1	0.174719	1.21	0.469707	1.074	310.34		WB
LAFQQGEARKF	Pol	6	A1	0.164966	1.275	0.428127	1.44	486.65		WB
WTVNDIQKL	Pol	407	A1 & C	0.157896	1.328	0.43906	1.333	432.35		WB
IATDIQTKEL	Pol	919	A1 & C	0.157511	1.331	0.356511	2.368	1056.17		WB
FVNTPPLVKLWY	Pol	571	A1 & C	0.155907	1.344	0.422651	1.501	516.35		WB

TQIGCTLNF	Pol	147	A1	0.152978	1.368	0.461041	1.136	340.84		WB
LVNQIIEKL	Pol	672	A1	0.149606	1.395	0.287707	3.771	2223.53		WB
YFSVPLDESF	Pol	270	A1	0.137998	1.502	0.467101	1.091	319.21		WB
ATWIPEWEF	Pol	563	A1 & C	0.13467	1.533	0.282179	3.918	2360.58		WB
ATDIQTKEL	Pol	920	A1 & C	0.133302	1.546	0.198066	7.368	5864.94		WB
ITKIQNFRVY	Pol	932	A1	0.121022	1.67	0.477253	1.024	286		WB
ETPGIRYQY	Pol	293	A1 & C	0.116612	1.721	0.178555	8.582	7243.45		WB
VVHTDNGSNF	Pol	827	A1	0.115483	1.736	0.324699	2.92	1490.11		WB
RSKNPEIIIY	Pol	327	A1	0.115474	1.736	0.305731	3.325	1829.57		WB
TAYFLLKL	Pol	812	A1	0.113739	1.759	0.277299	4.057	2488.57		WB
FSVPLDESFRKY	Pol	271	A1	0.112764	1.772	0.457089	1.166	355.73		WB
VYYDPSKDL	Pol	472	A1 & C	0.112341	1.777	0.245583	5.086	3507.38		WB
FPISPIETV	Pol	155	A1 & C	0.112292	1.778	0.328478	2.851	1430.41		WB
FFRENLAF	Pol	1	A1 & C	0.109798	1.811	0.36058	2.307	1010.68		WB
GGFIKVKQY	Pol	107	A1	0.109739	1.812	0.180556	8.441	7088.31		WB
HTDNGSNF	Pol	829	A1 & C	0.107207	1.845	0.230935	5.676	4109.73		WB
VTVLDVGDAY	Pol	261	A1 & C	0.106708	1.852	0.402489	1.714	642.22		WB
ILKDPVHGVYY	Pol	464	A1	0.106233	1.858	0.203807	7.03	5511.72		WB
SKIGPENPY	Pol	203	A1	0.104838	1.877	0.289602	3.721	2178.41		WB
KMIGGIGGF	Pol	101	A1 & C	0.104399	1.882	0.451857	1.208	376.45		WB
IQNFRVYY	Pol	935	CnA1	0.103473	1.895	0.230427	5.699	4132.38		WB
HQKEPPFLW	Pol	376	A1 & C	0.102662	1.906	0.158378	10.229	9010.66		WB
YALGIIQAQ	Pol	656	A1 & C	0.101253	1.925	0.408886	1.642	599.28		WB
TFSFPQITL	Pol	53	A1	0.097074	1.984	0.249974	4.93	3344.64		WB
FPQGEAREF	Pol	8	C	0.34726	0.584	0.406087	1.672	617.7		WB
IKIQNFRVY	Pol	929	C	0.21752	0.967	0.427914	1.442	487.77		WB

LVNQIIEQL	Pol	668	C	0.213079	0.986	0.343043	2.585	1221.86		WB
YTAFTIPSI	Pol	278	C	0.211829	0.991	0.620301	0.34	60.84		WB
TAYYILKL	Pol	808	C	0.205678	1.022	0.316606	3.083	1626.48		WB
KALTDIVPL	Pol	438	C	0.204854	1.027	0.546496	0.632	135.21		WB
WAGIQQEF	Pol	843	C	0.196719	1.072	0.356579	2.367	1055.4		WB
RANSPTSREL	Pol	22	C	0.185245	1.141	0.389929	1.873	735.71		WB
FSVPLDEGFRKY	Pol	267	C	0.16102	1.302	0.457413	1.164	354.49		WB
TLNFPQITL	Pol	49	C	0.159456	1.315	0.312476	3.175	1700.8		WB
GGFIKVRQY	Pol	103	C	0.1474	1.415	0.218402	6.253	4706.58		WB
ILKEPVHGVY	Pol	460	C	0.139875	1.485	0.314137	3.138	1670.51		WB
QLIKKERVY	Pol	675	C	0.133957	1.54	0.248639	4.973	3393.3		WB
KQIIKIQNF	Pol	926	C	0.131944	1.559	0.252557	4.846	3252.46		WB
ITKIGPENPY	Pol	198	C	0.1308	1.57	0.421384	1.514	523.48		WB
RAMASEFNL	Pol	731	C	0.121006	1.67	0.492241	0.919	243.19		WB
LKEPVHGVY	Pol	461	C	0.116511	1.722	0.312324	3.178	1703.61		WB
FRAQNPEIVIY	Pol	322	C	0.114352	1.75	0.276719	4.075	2504.24		WB
RAQNPEIVI	Pol	323	C	0.101652	1.92	0.337334	2.689	1299.71		WB
VPLDEGFRKY	Pol	269	C	0.100791	1.932	0.187678	7.98	6562.61		WB
KAVRIIKIL	Rev	14	A1	0.153297	1.365	0.371364	2.145	899.38		WB
RSAEPVPLQL	Rev	66	A1	0.142886	1.457	0.267964	4.35	2753.06		WB
AVRIIKILY	Rev	15	A1 & C	0.101483	1.922	0.204064	7.014	5496.41		WB
LQLPPIERL	Rev	73	C	0.14727	1.416	0.226179	5.889	4326.75		WB
QAVRIIKIL	Rev	14	C	0.132609	1.553	0.334777	2.736	1336.17		WB
RPAEPVPLQL	Rev	66	C	0.108441	1.829	0.204054	7.015	5497.01		WB
FLNKGLGISY	Tat	38	A1	0.229185	0.918	0.45994	1.144	344.93		WB
LNKGLGISY	Tat	39	A1	0.150828	1.385	0.288096	3.761	2214.19		WB

FQTKGLGISY	Tat	38	C	0.331945	0.618	0.50803	0.821	205		WB
NSLVKHHMY	Vif	22	A1 & C	0.315654	0.654	0.380199	2.012	817.38		WB
DARLVVRTY	Vif	61	A1	0.239778	0.878	0.29818	3.494	1985.32		WB
LADQLIHLHY	Vif	102	A1	0.211333	0.993	0.422226	1.505	518.73		WB
VVSPRCEY	Vif	128	A1	0.205318	1.024	0.294344	3.595	2069.46		WB
KTKPPLPSV	Vif	158	A1	0.195951	1.076	0.267004	4.381	2781.8		WB
LADQLIHLH	Vif	102	A1	0.183138	1.155	0.301829	3.412	1908.47		WB
YSTQIDPDL	Vif	94	A1	0.12009	1.679	0.465731	1.1	323.98		WB
HNKVGSLQY	Vif	139	A1 & C	0.115281	1.738	0.240839	5.269	3692.11		WB
LGHGVSIEW	Vif	81	A1 & C	0.110124	1.806	0.295045	3.576	2053.82		WB
LQYLALKAL	Vif	145	A1	0.108206	1.832	0.431922	1.398	467.07		WB
KAILGHIVI	Vif	122	C	0.227424	0.925	0.511344	0.801	197.78		WB
LADQLIHMHY	Vif	102	C	0.210579	0.996	0.442515	1.301	416.49		WB
FADSAIRKA	Vif	115	C	0.174186	1.213	0.39092	1.861	727.86		WB
YSTQVDPGL	Vif	94	C	0.171802	1.23	0.506153	0.832	209.21		WB
DARLVIKTY	Vif	61	C	0.168372	1.253	0.238014	5.379	3806.71		WB
LADQLIHMH	Vif	102	C	0.164259	1.28	0.281472	3.937	2378.71		WB
LQYLALTAL	Vif	145	C	0.158573	1.322	0.511877	0.798	196.64		WB
KIKPPLPSV	Vif	158	C	0.142395	1.461	0.222732	6.046	4491.16		WB
LADQLIHM	Vif	102	C	0.134856	1.532	0.265169	4.438	2837.58		WB
HIVIPRCDY	Vif	127	C	0.099586	1.949	0.365113	2.239	962.31		WB
RANGWFYRHHY	Vif	34	C	0.097793	1.973	0.331941	2.788	1377.81		WB
LGQHIYNTY	Vpr	42	A	0.243689	0.864	0.424712	1.478	504.96		WB
FPRPWLHGL	Vpr	34	A	0.182312	1.16	0.338313	2.671	1286.02		WB
AIIRTLQQL	Vpr	59	A	0.117168	1.713	0.283589	3.881	2324.84		WB
HSLGQYIY	Vpr	40	C	0.11724	1.712	0.270843	4.26	2668.62		WB

WTLELLEEL	Vpr	18	C	0.106043	1.861	0.445803	1.269	401.93		WB
LGQYIYETY	Vpr	42	C	0.103312	1.897	0.400359	1.742	657.2		WB
WTIVGIEY	Vpu	23	A	0.196678	1.072	0.360771	2.304	1008.6		WB
YAIVALVVAF	Vpu	7	A	0.183715	1.151	0.660056	0.234	39.57		WB
AIVALVVAF	Vpu	8	A	0.131763	1.561	0.356484	2.368	1056.48		WB
IVALVVAF	Vpu	9	A	0.125815	1.618	0.274184	4.155	2573.88		WB
YAIVALVV	Vpu	7	A	0.105075	1.873	0.438317	1.34	435.84		WB
MVDMGHLRL	Vpu	72	C	0.319918	0.645	0.497757	0.883	229.1		WB
AIVVWTIVY	Vpu	24	C	0.17373	1.217	0.378767	2.034	830.15		WB
IVYIEYRKL	Vpu	30	C	0.168865	1.249	0.345592	2.539	1188.62		WB
IVVWTIVY	Vpu	25	C	0.136583	1.516	0.3267	2.883	1458.2		WB
IAIVVWTIVY	Vpu	23	C	0.125981	1.617	0.542971	0.648	140.46		WB
YTPGPGTRFPLTF	Nef	128	A	f 0.097320	0 1.98	0 0.35952	8 2.32	3 1022.25		WB

Table S2. Stereochemical and spatial analysis of the HLA-C*03:02 model by different computational tools

Spatial analysis		Steriochemical analysis							Overall
Verify 3D	ProSA	ERRAT	PROVE	Pro-Check			ProQ	RAMPAGE	ProTSAV
				E	W	P			
99.63	-9.08	94.667	1.8	1	6	2	8.708	99.578	~0.525

Table S3. Hydrogen bond occupancy of C*03:02 residues making contact with peptides

Amino Acid Residue	Peptide			
	**$GY9_{71}$**	**$AY9_{324}$**	**$GF10_{43}$**	**$VL9_{109}$**
Y8	0.00%	88.73%	0.00%	0.00%
E62-1	88.79%	94.43%	87.10%	64.06%
E62-2	84.76%	92.40%	0.00%	0.00%
E62-3	0.00%	91.09%	0.00%	0.00%
K65	61.87%	69.68%	91.77%	0.00%
R68	54.58%	0.00%	0.00%	0.00%
Y83	74.37%	0.00%	80.85%	0.00%
R96	0.00%	0.00%	0.00%	73.74%
Y98	0.00%	84.12%	0.00%	0.00%
N113	91.48%	0.00%	0.00%	0.00%
T142	67.90%	66.70%	59.10%	94.36%
K145-1	67.35%	56.30%	58.07%	50.00%
K145-2	54.80%	52.26%	0.00%	0.00%
W146-1	93.04%	73.91%	73.25%	0.00%
W146-2	65.79%	0.00%	0.00%	0.00%
E151-1	95.74%	0.00%	53.94%	59.68%
E151-2	92.87%	0.00%	0.00%	0.00%
E151-3	80.76%	0.00%	0.00%	0.00%
Y158	0.00%	61.57%	0.00%	0.00%

Table S4. Calculated MM-PBSA binding energy difference

Amino acid mutation	Binding Free Energy Difference			
	$GY9_{71}$	$AY9_{324}$	$GF10_{43}$	$VL9_{109}$
E62A	-25.54	-47.47	-11.91	-9.25
E151A	-23.4	3.93	-6.47	-1.98
T142A	-12.97	-9.94	-8.77	-9.91
N113A	-11.49	2	-2.64	-9.27
K145A	-10.8	-1.54	-6.9	-7.65
S115A	-10.66	-0.09	-2.63	0.8
W146A	-8.55	-4.03	-5.13	-5.51
Y83A	-7.98	-0.16	-4.84	0.07
R68A	-6.36	-0.49	-0.06	-15.49
K65A	-6.2	-2.05	-5.08	-6.33
N79A	-3.12	1.37	-0.57	1.43
S76A	-2.57	1.03	-1.35	0.62
L155A	-2.11	1.62	0.21	3.28
R96A	-1.78	0.73	1.68	-32.25
L80A	-1.64	0.33	-1.62	0.17
I123A	0.53	-0.91	-1.62	0.16
Y158A	0.43	-3.51	-1.59	-5.99
Y122A	-1.44	-0.87	-1.13	-1.16
V75A	-0.11	0.14	-0.65	0.25
Y98A	2.53	-0.13	-0.38	0.48
R61A	-0.41	0.01	-0.26	0.23
F32A	-0.22	-0.05	-0.14	-0.53
Y8A	4.19	4.21	-0.13	-5.36
L94A	1.22	-0.16	-0.13	3.05
M4A	-0.49	-0.35	-0.1	-0.56
Y170A	2.91	0.75	-0.06	0.94
Q154A	0.02	0.3	-0.04	0.16
Q95A	0.03	-0.05	-0.01	0.03
Y58A	2.81	0.74	0.11	-1.04
I141A	0.31	0.12	0.3	0.3
T72A	-1.45	-0.37	0.56	-1.4
Y6A	1.7	-0.82	0.64	-2.69
W166A	1.21	2.39	1.16	0.8
Y66A	2.93	0.53	1.25	-3.58
Q69A	0.73	3.13	1.53	0.78

All values are given in kcal/mol

Table S5. Participant characteristics and IFN-γ response

No.	Age (Yrs)	Sex	ART Duration (Mo)	Progression status	HLA locus						IFN-γ response (SFU/10^6 cells)	HIV-1 Clade
					A		B		C			
**HLA-C*03:02 positive**												
P1	2.3	Female	18	RP	A*31:01	A*23:01	B*82:02	B*08:01	C*07:01	C*03:02	10	D
P2	13.9	Male	30	LTNP	ND	ND	B*81:01	B*45:01	C*03:02	C*18:01	940	A1
P3	20.8	Female	35	LTNP	A*30:01	A*02:01	B*42:01	B*58:01	C*03:02	C*17:01	65	C
P4	15.5	Male	4	LTNP	A*74:01	A*74:01	B*35:01	B*58:01	C*04:01	C*03:02	0	No DNA
P5	14.3	Male	45	LTNP	A*30:02	A*31:04	B*15:64	B*58:01	C*07:01	C*03:02	5	A1
P6	12.0	Female	1	LTNP	A*03:01	A*68:02	B*82:02	B*45:01	C*16:01	C*03:02		No DNA
P7	6.4	Female	68	RP	A*02:02	A*02:01	B*58:01	B*49:01	C*07:01	C*03:02	15	A1D
P8	3.0	Female	15	RP	A*74:01	A*30:02	B*58:01	B*45:01	C*06:02	C*03:02		A1D
P9	2.5	Female	8	RP	A*74:01	A*23:01	B*07:02	B*58:01	C*07:02	C*03:02	385	A1
P10	9.6	Male	104	RP	A*30:01	A*74:01	B*82:02	B*57:03	C*03:02	C*07:01		A1
P11	14.1	Female	26	LTNP	A*30:02	A*23:01	B*45:01	B*58:01	C*03:02	C*06:02	5	A1
P12	14.3	Female	35	LTNP	A*30:02	A*29:02	B*39:10	B*82:02	C*12:03	C*03:02	10	D
P13	11.3	Male	2	LTNP	A*74:01	A*23:01	B*07:02	B*58:01	C*07:02	C*03:02	15	No DNA
**HLA-C*03:02 negative**												
C1	12.5	Female	121	RP	A*29:02	A*23:01	B*42:01	B*45:01	C*06:30	C*17:11	0	
C2	11.5	Female	1	LTNP	A*26:12	A*02:05	B*58:02	B*57:03	C*07:01	C*06:02	5	
C3	13.6	Female	1	LTNP	A*03:01	A*68:02	B*15:10	B*15:03	C*02:27	C*03:07	5	
C4	18.6	Female	8	LTNP	A*74:01	A*02:01	B*53:01	B*44:15	C*04:01	C*04:07	0	
C5	3.2	Male	35	RP	A*66:01	A*29:02	B*42:01	B*58:02	C*06:30	C*17:11	0	
C6	17.5	Female	1	LTNP	A*34:02	A*30:01	B*42:01	B*47:03	C*17:01	C*07:01	0	
C7	12.9	Male	1	LTNP	A*68:02	A*32:01	B*81:01	B*15:10	C*03:04	C*08:04	5	
C8	10.9	Female	1	LTNP	A*74:01	A*23:01	B*15:03	B*15:03	C*02:10	C*02:10	5	
C9	10.6	Male	101	RP	A*30:02	A*26:12	B*15:03	B*57:02	C*02:10	C*18:01	15	
C10	6.3	Male	59	RP	A*30:02	A*68:02	B*42:01	B*49:01	C*17:01	C*07:01	15	

C11	16.4	Male	1	LTNP	A*29:02	A*02:10	B*15:16	B*45:01	C*14:02	C*06:02	20
C12	6.7	Male	53	RP	A*34:02	A*68:01	B*35:71	B*44:37	C*07:01	C*07:05	5

Abbreviations: ND, not detected; SFU, spot forming units
